# Inactivation of PNKP by Mutant ATXN3 Triggers Apoptosis by Activating the DNA Damage-Response Pathway in SCA3

**DOI:** 10.1371/journal.pgen.1004834

**Published:** 2015-01-15

**Authors:** Rui Gao, Yongping Liu, Anabela Silva-Fernandes, Xiang Fang, Adriana Paulucci-Holthauzen, Arpita Chatterjee, Hang L. Zhang, Tohru Matsuura, Sanjeev Choudhary, Tetsuo Ashizawa, Arnulf H. Koeppen, Patricia Maciel, Tapas K. Hazra, Partha S. Sarkar

**Affiliations:** 1 Department of Neurology, University of Texas Medical Branch, Galveston, Texas, United States of America; 2 Life and Health Sciences Research Institute (ICVS), School of Health Sciences, University of Minho, Braga, Portugal; 3 ICVS/3B’s PT Government Associate Laboratory, Braga/Guimarặes, Portugal; 4 Department of Biomedical Engineering, University of Texas Medical Branch, Galveston, Texas, United States of America; 5 Department of Internal Medicine, University of Texas Medical Branch, Galveston, Texas, United States of America; 6 Department of Neurology, Jichi Medical School, Shimotsuke, Japan; 7 Department of Neurology and McNight Brain Research Institute, University of Florida, Gainesville, Florida, United States of America; 8 Department of Neurology, Albany Stratton VA Medical Center, Albany, New York, United States of America; 9 Neuroscience and Cell Biology, University of Texas Medical Branch, Galveston, Texas, United States of America; The Hospital for Sick Children and University of Toronto, CANADA

## Abstract

Spinocerebellar ataxia type 3 (SCA3), also known as Machado-Joseph disease (MJD), is an untreatable autosomal dominant neurodegenerative disease, and the most common such inherited ataxia worldwide. The mutation in SCA3 is the expansion of a polymorphic CAG tri-nucleotide repeat sequence in the C-terminal coding region of the *ATXN3* gene at chromosomal locus 14q32.1. The mutant ATXN3 protein encoding expanded glutamine (polyQ) sequences interacts with multiple proteins *in vivo*, and is deposited as aggregates in the SCA3 brain. A large body of literature suggests that the loss of function of the native ATNX3-interacting proteins that are deposited in the polyQ aggregates contributes to cellular toxicity, systemic neurodegeneration and the pathogenic mechanism in SCA3. Nonetheless, a significant understanding of the disease etiology of SCA3, the molecular mechanism by which the polyQ expansions in the mutant ATXN3 induce neurodegeneration in SCA3 has remained elusive. In the present study, we show that the essential DNA strand break repair enzyme PNKP (polynucleotide kinase 3’-phosphatase) interacts with, and is inactivated by, the mutant ATXN3, resulting in inefficient DNA repair, persistent accumulation of DNA damage/strand breaks, and subsequent chronic activation of the DNA damage-response ataxia telangiectasia-mutated (ATM) signaling pathway in SCA3. We report that persistent accumulation of DNA damage/strand breaks and chronic activation of the serine/threonine kinase ATM and the downstream p53 and protein kinase C-δ pro-apoptotic pathways trigger neuronal dysfunction and eventually neuronal death in SCA3. Either PNKP overexpression or pharmacological inhibition of ATM dramatically blocked mutant ATXN3-mediated cell death. Discovery of the mechanism by which mutant ATXN3 induces DNA damage and amplifies the pro-death signaling pathways provides a molecular basis for neurodegeneration due to PNKP inactivation in SCA3, and for the first time offers a possible approach to treatment.

## Author Summary

Spinocerebellar ataxia type 3 (SCA3) is an untreatable neurodegenerative disease, and the most common dominantly inherited ataxia worldwide. SCA3 is caused by expansion of a CAG tri-nucleotide repeat sequence in the *ATXN3* gene’s coding region. The expanded CAG sequences encode a run of the amino acid glutamine; the mutant ATXN3 interacts with multiple proteins *in vivo* to create insoluble aggregates in SCA3 brains. It is thought that the loss of function of the aggregated proteins contributes to cellular toxicity and neurodegeneration in SCA3. Despite significant progress in understanding SCA3’s etiology, the molecular mechanism by which the mutant protein triggers the death of neurons in SCA3 brains remains unknown. We now report that the mutant ATXN3 protein interacts with and inactivates PNKP (polynucleotide kinase 3’-phosphatase), an essential DNA strand break repair enzyme. This inactivation results in persistent accumulation of DNA damage, and chronic activation of the DNA damage-response ATM signaling pathway in SCA3. Our work suggests that persistent DNA damage/strand breaks and chronic activation of ATM trigger neuronal death in SCA3. Discovery of the mechanism by which mutant ATXN3 induces DNA damage and amplifies the pro-death pathways provides a molecular basis for neurodegeneration in SCA3, and perhaps ultimately for its treatment.

## Introduction

Spinocerebellar ataxia type 3 (SCA3), also known as Machado-Joseph disease (MJD), is an autosomal dominant neurodegenerative disease caused by CAG repeat expansion in the C-terminal coding region of the *ATXN3* gene [1–3]. SCA3 is the most common dominantly inherited ataxia world-wide, and a late-onset disease that manifests with cerebellar ataxia, peripheral nerve palsy, and pyramidal and extrapyramidal signs [1–4]. SCA3 neurodegeneration is primarily observed in the brainstem, cerebellum, basal ganglia and spinal cord [5–8]. Ataxia symptoms appear between the ages of 20 and 50 years, and manifest with cerebellar ataxia, opthalmoplegia, dysarthria, dysphagia, dystonia, rigidity and distal muscle atrophies [1–3, 8, 9]. The wild-type *ATXN3* gene encodes 12 to 41 CAG repeats in its 10^th^ exon at the human chromosomal locus 14q32.1 [3]. ATXN3 is a deubiquitinating enzyme that edits specific poly-ubiquitin linkages [10, 11]. It has also been linked to transcriptional regulation [9, 12]. However, ATXN3 does not seem to be essential for brain development and function, as mice lacking ATXN3 do not develop overt neurological phenotypes [13]. Therefore, the exact function of ATXN3 remains unknown, limiting efforts to establish the possible role of mutant ATXN3 in eliciting neuronal death in SCA3. In SCA3, the polymorphic CAG repeats are expanded to 62 to 84 glutamines and the mutant ATXN3 forms aggregates that are deposited in SCA3 neurons [2, 3]. A large body of literature supports the hypothesis that multiple proteins aberrantly interact with the mutant ATXN3 and that the loss of function of the mutant ATXN3-interacting proteins contributes to neurodegeneration and SCA3 pathology [2, 8–9]. Recent studies have reported that depletion of the mutant ATXN3 allele in a SCA3 transgenic mouse brains rescues the molecular phenotypes of SCA3 supporting the hypothesis that mutant ATXN3 elicits toxicity and neuronal dysfunction in SCA3 [14]. Recent studies have also shown that the mutant ATXN3 causes p53-mediated neuronal death *in vitro* and *in vivo* by activating the transcription of the p53-inducibe pro-apoptotic genes such as *BAX* (Bcl2-associated X protein) and *PMAIP1* (PUMA, p53 upregulated modulator of apoptosis), triggering mitochondrial apoptotic pathways [15, 16]. However, the mechanism by which mutant ATXN3 increases p53 phosphorylation and activates the p53-dependent pro-apoptotic signaling pathways to facilitate neuronal death and dysfunction remains unknown.

In the present study we show that PNKP (Polynucleotide kinase 3’-phosphatase), a dual- function DNA strand break repair enzyme [17, 18], is a native ATXN3-interacting protein, and is inactivated by its interaction with the mutant ATXN3 in SCA3. Our data also show that PNKP is also present, in part in the polyQ aggregates in SCA3 brain. Diminished PNKP activity results in persistent accumulation of DNA strand breaks, leading to chronic activation of the DNA damage-response ataxia telangiectasia mutated (ATM) protein kinase and the downstream pro-apoptotic p53-dependent signaling pathways in SCA3. Additionally, activated ATM stimulates phosphorylation of c-Abl tyrosine kinase, which phosphorylates and facilitates nuclear inclusion of protein kinase C delta (PKCδ), further amplifying pro-apoptotic output in SCA3. Either overexpression of PNKP or pharmacological inhibition of ATM in mutant ATXN3-expressing cells blocked aberrant activation of the pro-death pathways and reduced cell death, suggesting that mutant ATXN3-mediated chronic activation of the DNA damage-response ATM signaling pathway plays a pivotal role in neuronal dysfunction and neurodegeneration in SCA3. Therefore, our current study not only provides an insight into the mechanism of neurodegeneration in SCA3, but also delineates potential drug targets for developing mechanism-based efficacious therapeutic modalities to combat systemic degeneration of neuronal cells in SCA3.

## Results

### PNKP interacts with the mutant ATXN3 and is sequestered into the ATXN3-polyQ aggregates in SCA3 brain

**Figure 1:**
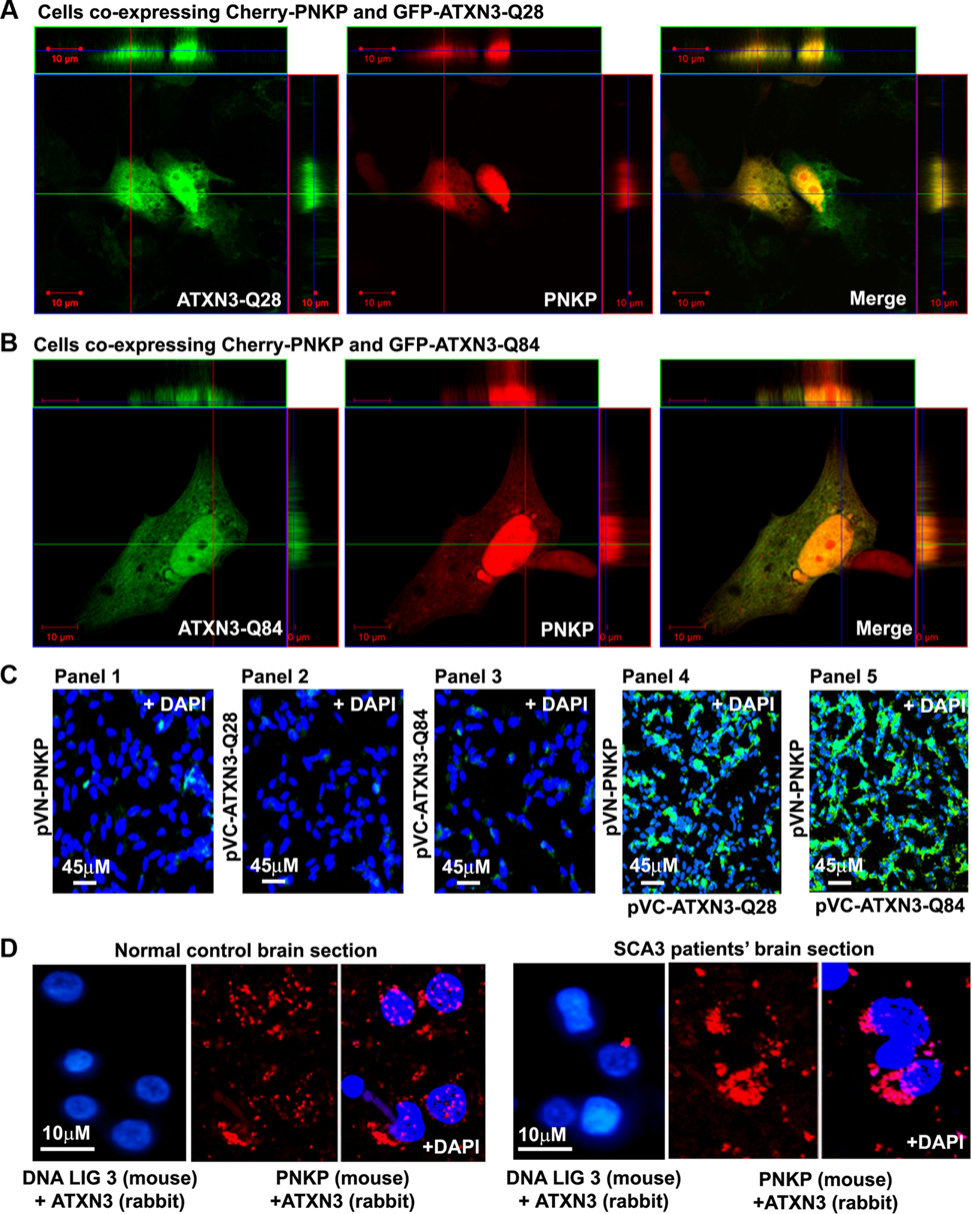
PNKP interacts with both wild-type and mutant ATXN3 in cells and human brain sections. **(A)** Plasmids pGFP-ATXN3-Q28 and pCherry-PNKP were co-transfected into SH-SY5Y cells and co-localization of PNKP and ATXN3-Q28 was assessed by confocal microscopy; the merge of green and red fluorescence from ATXN2-Q28 and PNKP, respectively, appears as yellow/orange fluorescence. Nuclei were stained with DAPI in C-D. **(B)** Plasmids pGFP-ATXN3-Q84 and pCherry-PNKP were co-transfected into SH-SY5Y cells and co-localization of PNKP and ATXN3-Q84 was assessed as in A. **(C)** Bimolecular fluorescence complementation assay. SH-SY5Y cells were transfected with plasmid: Panel 1) pVN173-PNKP; Panel 2) pVC155-ATXN3-Q28; Panel 3) pVC155-ATXN3-Q84; Panel 4) co-transfected with pVN173-PNKP and pVC155-ATXN3-Q28; or Panel 5) pVN173-PNKP and pVC155-ATXN3-Q84. Reconstitution of green/yellow fluorescence was assessed 48 hours after transfection using fluorescence microscopy (20X). Nuclei were stained with DAPI in Figs. 1C and 1D. **(D)** Proximity ligation assays (PLAs) were performed on control and SCA3 human brain sections, using mouse anti-PNKP and rabbit anti-ATXN3 primary antibodies, and anti-DNA ligase 3 (DNA LIG3) (rabbit) with anti-ATXN3 (mouse) antibodies as control. The generation of red fluorescence was monitored under a fluorescence microscope.

**Figure 2:**
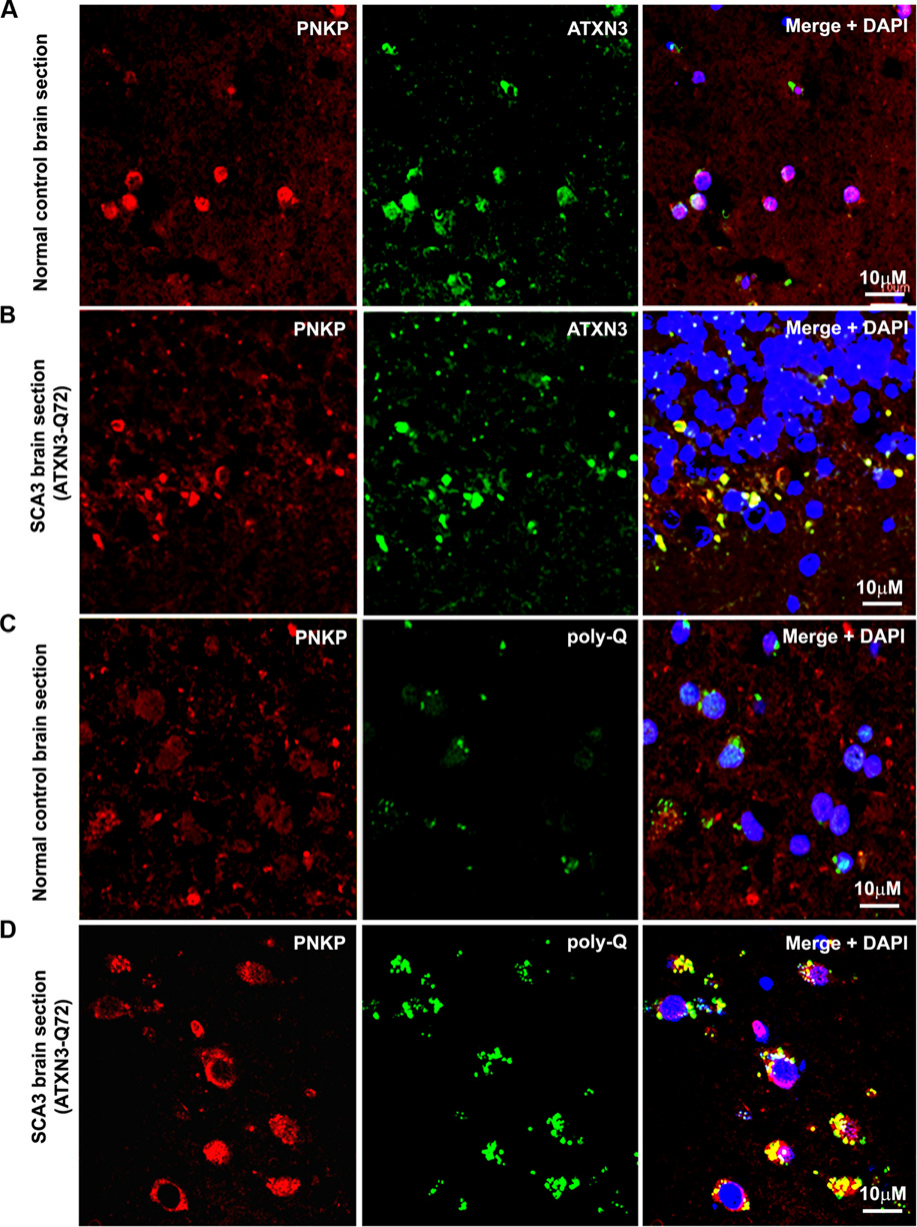
PNKP co-localizes with wild-type and mutant ATXN3 in human brain sections. **(A)** Normal human brain sections were analyzed by immunostaining with anti-PNKP (red) and anti-ATXN3 (green) antibodies to assess *in vivo* interaction of PNKP and ATXN3; merge of red and green fluorescence appears as yellow/orange fluorescence, Nuclei were stained with DAPI in Figs. 1A to 1D; **(B)** SCA3 (expressing mutant ATXN3-Q72) brain sections were analyzed by immunostaining with anti-PNKP (red), and anti-ATXN3 (green) antibodies to assess *in vivo* interaction of PNKP and ATXN3; merge of red and green fluorescence appears as yellow/orange fluorescence; **(C)** Normal human brain sections were analyzed by immunostaining with anti-PNKP (red) and anti-polyQ antibody (green) antibodies to assess the presence of PNKP in the polyQ aggregates; **(D)** SCA3 (expressing ATXN3-Q72) brain sections were analyzed by immunostaining with anti-PNKP (red), and anti-polyQ (green) antibodies to determine the presence of PNKP in the polyQ aggregates; merge of red and green fluorescence appears as yellow/orange fluorescence.

**Figure 3:**
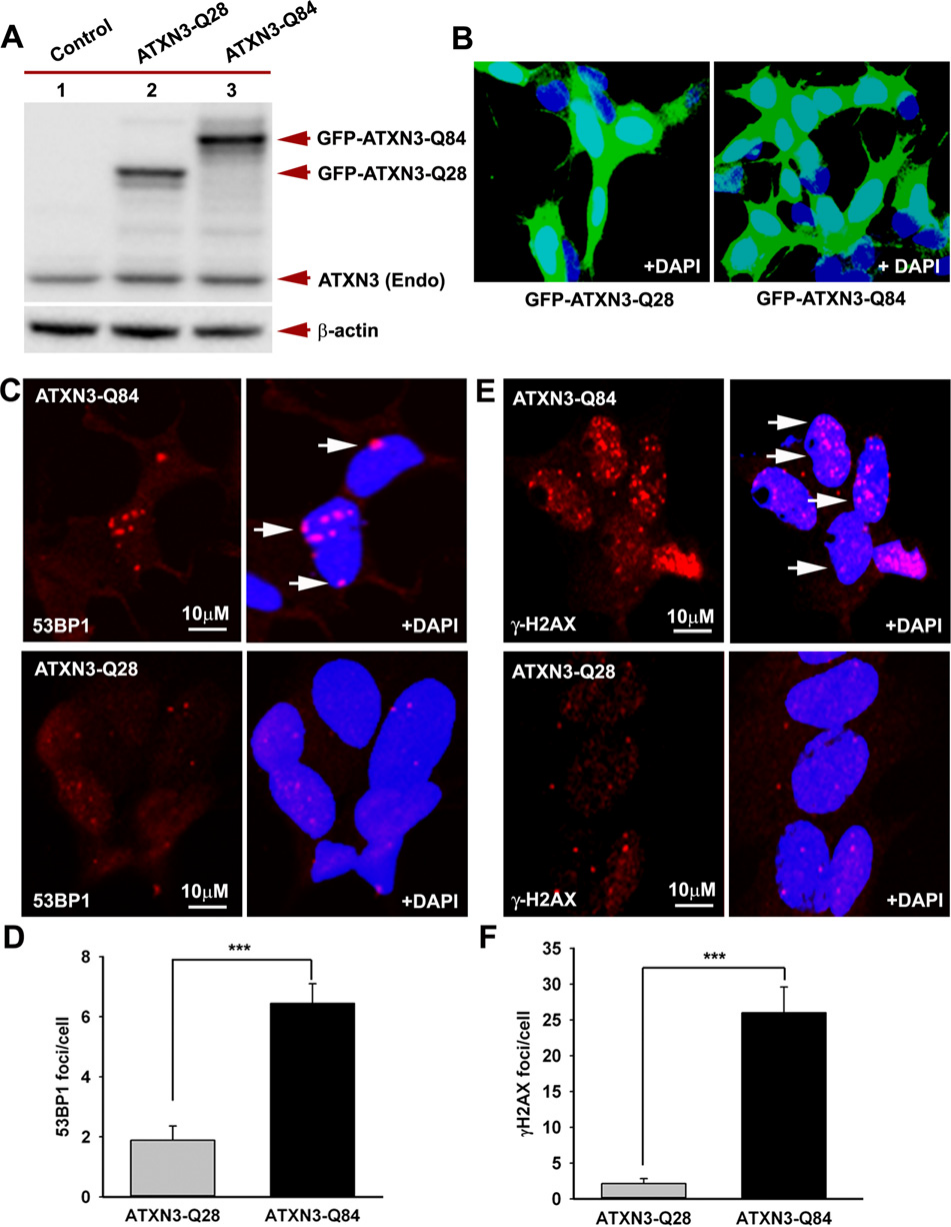
Expression of mutant ATXN3 in cells induces DNA strand breaks. **(A)** Expression of GFP-ATXN3-Q84 and GFP-ATXN3-Q28 was induced in SH-SY5Y cells; 48 hours after induction, the cells were harvested and their lysates analyzed by Western blotting with anti-ATXN3 monoclonal antibody to detect endogenous ATXN3 and exogenous GFP-ATXN3-Q28 and GFP-ATXN3-Q84 levels (shown by arrows). Lane 1, control SH-SY5Y cells; lane 2, SH-SY5Y cells expressing GFP-ATXN3-Q28; lane 3, SH-SY5Y cells expressing ATXN3-Q84; β-actin was used as loading control. **(B)** Confocal images showing expression of GFP-tagged ATXN3-Q28 and ATXN3-Q84 in SH-SY5Y cells. Nuclei were stained with DAPI in B, C and E. **(C)** Expression of either GFP-ATXN3-Q28 or GFP-ATXN3-Q84 was induced and the cells analyzed by immunostaining with anti-p-53BP1-S1778 antibody (red) to assess DNA strand breaks; 53BP1 foci are shown by arrows. **(D)** Relative number of 53BP1 foci in the SH-SY5Y cells expressing ATXN3-Q28 or ATXN3-Q84 (n = 100, data represent mean ± SD, *** = p < 0.001). **(E)** SH-SY5Y cells expressing either ATXN3-Q84 or ATXN3-Q28 analyzed by immunostaining with anti-γH2AX-S139 antibody (red); γH2AX foci are shown by arrows. **(F)** Relative number of γH2AX foci in SH-SY5Y cells expressing ATXN3-Q28 or ATXN3-Q84 (n = 100, data represents mean ± SD, *** = p < 0.001).

Our studies described in the accompanying manuscript by Chatterjee et al suggest that PNKP is a native ATXN3-interacting protein, and that ATXN3 modulates PNKP activity and DNA repair (Chatterjee et al, Figs. 1–3). Immunoprecipitation of PNKP from the nuclear extract from human neuroblastoma SH-SY5Y cells and subsequent mass spectrometric analysis showed the presence of ATXN3 in the immunoprecipitated (IP) pellet; conversely, immunoprecipitation of ATXN3 and Western blot analysis revealed the presence of PNKP in the ATXN3 IP (Chatterjee et al, Figs. 1, S1, 2A and 2B). Further, GST pull-down from the nuclear extract, followed by Western blot analysis, indicated that both wild-type and mutant ATXN3 directly interact with PNKP *in vitro*, (Chatterjee et al; Fig. 2D). The wild-type ATXN3 protein stimulated, and in contrast, the mutant ATXN3 dramatically diminished, the 3’ phosphatase activity of PNKP *in vitro* (Chatterjee et al; Figs. 3A and 3B). The interaction between these two proteins was further validated in SH-SY5Y cells co-transfected with the plasmids pCherry-PNKP and pGFPC-ATXN3–28, expressing cherry-tagged PNKP and GFP-tagged ATXN3-Q28, respectively, and imaged by confocal microscopy. Analysis of the transfected cells showed significant co-localization of the red fluorescence of PNKP with the green fluorescence of ATXN3-Q28 (Fig. 1A). Similarly, cells co-transfected with pCherry-PNKP and pGFP-ATXN3-Q84 (a plasmid expressing mutant ATXN3-Q84 encoding 84 glutamines) showed marked co-localization of PNKP and ATXN3-Q84 (Fig. 1B). However, co-transfection of plasmid pCherry-PNKP and pAcGFPC1 (an empty control vector expressing GFP) did not show any detectable reconstitution of yellow/orange fluorescence (S1 Fig.), suggesting specificity of these interactions. Together, these data support our previous interpretation that both wild-type and mutant ATXN3 interact with PNKP in the cell (Chatterjee et al).

To further confirm the interaction of PNKP and ATXN3 *in cell*, we performed bi-molecular fluorescence complementation (Bi-FC) assays, a versatile method to assess *in cell* protein-protein interactions [19]. We cloned PNKP cDNA with the N-terminal amino acids of modified GFP into plasmid pBiFC-VN173, and ATXN3 cDNA (encoding 28 and 84 glutamines) with the C-terminal amino acids of modified GFP into plasmid pBiFC-VC155 (a description of these Bi-FC plasmids is provided in the Methods section). Transfection of pVN173-PNKP, pVC155-ATXN3-Q28 or pVC155-ATXN3-Q84 into SH-SY5Y cells individually did not reconstitute green/yellow fluorescence (Fig. 1C, panels 1–3). In contrast, co-transfection of plasmids pVN173-PNKP and pVC155-ATXN3-Q28 effectively reconstituted green/yellow fluorescence (Fig. 1C, panel 4). Importantly, co-transfection of plasmids pVN173-PNKP and pVC155-ATXN3-Q84 also resulted in robust reconstitution of green/yellow fluorescence (Fig. 1C, panel 5). These data substantiate our interpretation that both wild-type and mutant ATXN3 interact with PNKP in the cell. Furthermore, we analyzed these protein-protein interactions in SCA3 patients’ brain sections by proximity ligation assays (PLA), a widely used technique to assess *in vivo* protein-protein interactions [20]. The PLA analysis clearly shows a robust reconstitution of red fluorescence in both SCA3 and normal control brains, suggesting an *in vivo* interaction between ATXN3 and PNKP (n = 3; Fig. 1D). Importantly, about 70% of the ATXN3-PNKP complexes were detected in the nuclei in the control brain sections (Fig. 1D; panel 2 and 3). By contrast, PLA analysis of the SCA3 patients’ brain sections shows that the ATXN3-PNKP complexes are predominantly present in periphery or outside the nuclei (n = 3, Fig. 1D). Since PNKP is present in the mitochondria [18], the extra-nuclear signals detected in the control brain sections presumably are from the PNKP-ATXN3 complexes present in the mitochondria. To further verify the specificity of the *in vivo* interaction of ATXN3 and PNKP, we performed PLA analysis to check the interaction of ATXN3 with DNA ligase 3α (DNA LIG 3α), another critical DNA strand break repair enzyme present in the PNKP complex (Chatterjee et al; Figs. 2A, 2B and S2). The PLA analysis showed no significant interaction of ATXN3 with DNA LIG 3α in the brain sections from SCA3 patients, or in control brains under identical experimental conditions (Fig. 1D; panels 1 and 4), suggesting specificity of the interactions between ATXN3 and PNKP *in vivo*. Consistent with these data, PLA analysis also suggested specific and pronounced interactions of ATXN3 and PNKP in SH-SY5Y cells (accompanying manuscript, Chatterjee et al, Fig. 2C).

The *in vivo* interaction of PNKP and ATXN3 in SCA3 and control brain sections was further confirmed by immunostaining brain sections from SCA3 patients and control subjects with specific antibodies, followed by confocal microscopy. For immunostaining the brain sections we used an anti-PNKP antibody that shows high specificity for PNKP as evidenced by Western blot and immunohistochemical analyses (S2 Fig.). Image analysis revealed a distinct co-localization of PNKP with ATXN3 in the cerebellum of normal control brain and SCA3 (expressing mutant ATXN3 with 72 glutamines, ATXN3-Q72) brain sections (Figs. 2A and 2B). Likewise, analysis of SCA3 brain expressing either ATXN3-Q79 or ATXN3-Q84 also showed discrete co-localization of ATXN3 with PNKP (S3 Fig.). Importantly, consistent with the PLA data as described in Fig. 1D, majority (70 to 80%) of the ATXN3-PNKP complexes were detected in the periphery and/or outside of nuclei in the SCA3 brain sections (Figs. 2B and S3); in contrast, the ATXN3-PNKP complexes were predominantly detected inside the nuclei in the control brain sections (n = 3; Figs. 2A and S3). We next assessed *in vivo* interactions of ATXN3 and PNKP in transgenic mouse brains expressing mutant ATXN3, as well as in wild-type control mouse brains. Previous studies have shown that this novel SCA3 mouse model (CMVMJD135) develops SCA3-like motor incoordination and exhibits neurodegeneration in spite of the absence of aberrant cleavage of the mutant ATXN3 and accumulation of polyQ aggregates in brain [21]. We observed a distinct co-localization of PNKP with ATXN3 in the deep cerebellar nuclei area of the SCA3 mouse brain, as well as in age-matched control brain sections (S4 Fig.). To assess whether PNKP is present in the polyQ aggregates in SCA3 patients’ brains, brain sections from SCA3 and age-matched normal subjects were co-immunostained with an anti-polyQ antibody (5TF1–1C2; green) and anti-PNKP antibody (red). Confocal image analysis showed distinct co-localization of PNKP- and polyQ aggregates in brain sections from SCA3 patients, but not in control brain sections (Figs. 2C and 2D). The much less intense fluorescence signals observed in the brain sections from control subjects (Fig. 2C) presumably are from the shorter polyQ sequences present in the wild-type proteins, and do not show detectable co-localization with PNKP. Collectively, these data suggest that PNKP is a native wild-type ATXN3-interacting protein that also interacts with the mutant ATXN3, and at least in part is recruited into the polyQ aggregates in the SCA3 brain.

### Mutant ATXN3 induces genomic DNA damage in SCA3

Recent studies have shown elevated levels of DNA damage/strand breaks in peripheral blood lymphocytes in SCA3 patients [22], indicating that mutant ATXN3 can induce DNA damage. Our data in the accompanying manuscript by Chatterjee et al suggest that mutant ATXN3 binds PNKP and inhibits its 3’-phosphatase activity (Chatterjee et al, Figs. 2D and 3B). We thus examined SH-SY5Y cells expressing ATXN3-Q84, brain sections from SCA3 patients, as well as SCA3 transgenic mouse brains, for the presence of DNA damage. Oxidative DNA damage or double-strand breaks rapidly induce the phosphorylation of several damage-response kinases and thereby facilitate protein-protein interactions that collectively regulate a signaling cascade to repair DNA lesions. ATM is one of the major DNA damage-response kinases, and is rapidly activated by autophosphorylation at S1981 after genomic damage [23]. Genomic DNA damage or strand breaks also result in rapid phosphorylation of histone H2AX and p53-binding protein 1 (53BP1), and the phosphorylated H2AX (γH2AX) and 53BP1 (p-53BP1) are quickly recruited into damage sites and visualized as nuclear foci in damaged cells [23]. Since mutant ATXN3 interacts with and inactivates PNKP, we next investigated whether ectopic expression of mutant ATXN3 induces DNA damage. To this end we developed SH-SY5Y cells expressing ATXN3-Q28 and ATXN3-Q84; Western blotting and confocal image analysis confirmed effective expression of the wild-type ATXN3-Q28 as well as mutant ATXN3-Q84 in these cells (Figs. 3A and 3B). To assess the accumulation of genomic DNA damage in cells expressing mutant ATXN3, we used anti-phospho-53BP1 (p-53BP1-S1778) antibody to perform immunohistochemical (IHC) analysis on the SH-SY5Y cells expressing wild-type vs. mutant ATXN3. We observed about a 5-fold increase in 53BP1 focus formation in the SH-SY5Y cells expressing ATXN3-Q84 over the cells expressing ATXN3-Q28 (Figs. 3C and 3D; the foci are shown by arrows). IHC analysis also revealed significantly more (~10-fold) γH2AX foci in the mutant ATXN3-expressing cells compared to control cells (Figs. 3E and 3F). Consistent with these results, immunohistological analysis of SCA3 brain sections with phospho-53BP1 (p-53BP1-S1778) antibody also showed widespread 53BP1 nuclear foci, unlike controls (n = 3) (S5 Fig.; the foci are shown by arrows). Further, comet analysis of neuronal cells from the deep cerebellar nuclei from a SCA3 transgenic mouse brain revealed the presence of DNA damage (S6A and S6B Figs.). Compared to control cells, DNA damage in the mutant cells was significantly increased when exposed to hydrogen peroxide (S6C, S6D and S6E Figs.). To further test our hypothesis that sequestration of PNKP can compromise the cellular DNA damage repair ability, resulting in increased accumulation of DNA lesions, we depleted PNKP in SH-SY5Y cells with siRNA. Western blotting confirmed about 70 to 80% depletion of PNKP in the *PNKP-siRNA*-treated cells (S7A and S7B Figs.). These cells showed about a 5-fold increase in the formation of 53BP1 foci compared to the *control siRNA*-treated cells (S7C and S7D Figs.). Likewise, the *PNKP*-depleted cells also showed about 10-fold more γH2AX focus formation vs. cells transfected with *control-siRNA* (S7E and S7F Figs.). These data clearly suggest that the perturbation of PNKP activity by the mutant ATXN3 impairs DNA repair efficacy and facilitates the accumulation of DNA strand breaks in SCA3.

### Mutant ATXN3 activates the DNA damage-response pathway in SCA3

**Figure 4:**
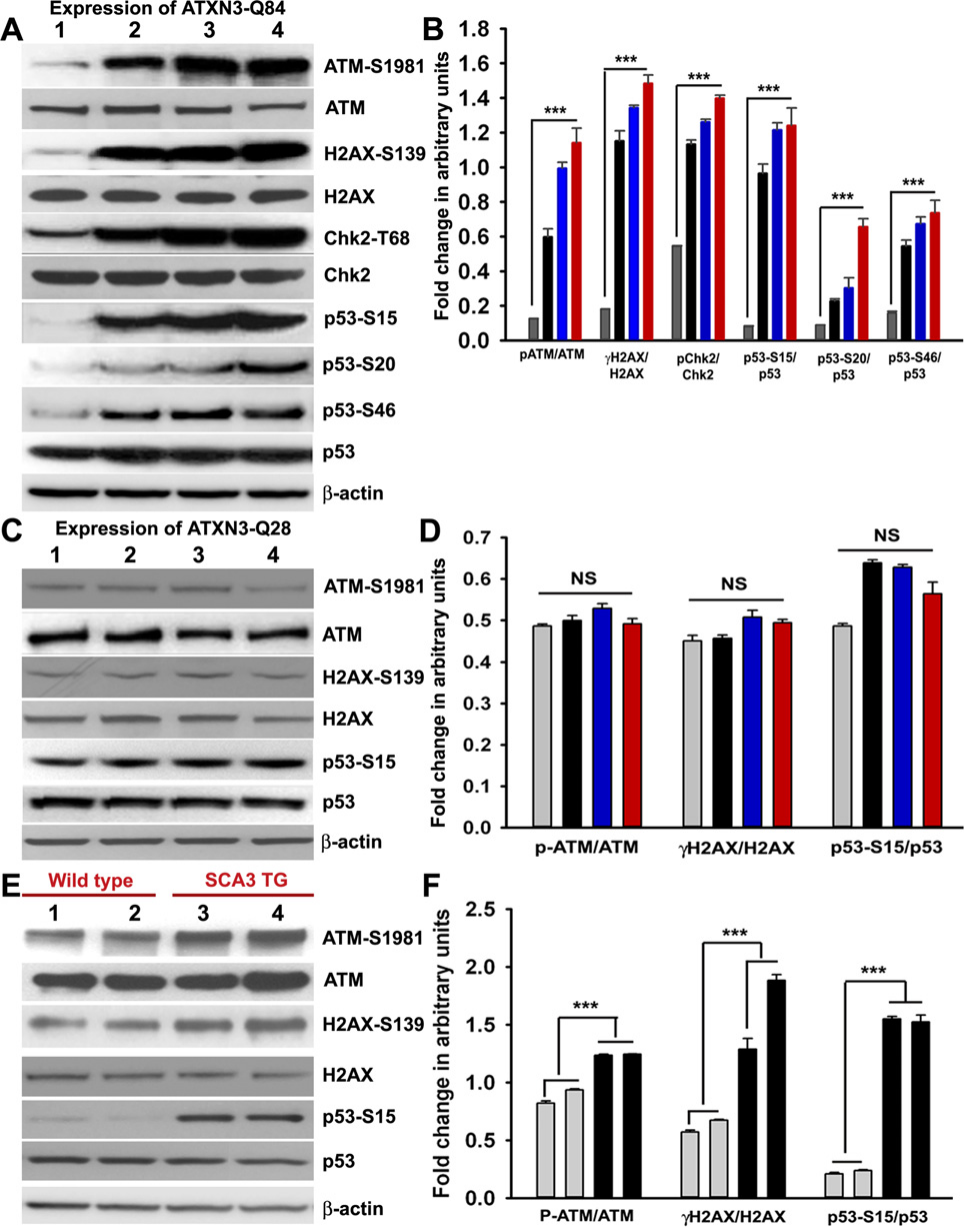
Mutant ATXN3 activates DNA damage-response *in vitro* and *in vivo*. **(A)** Expression of ATXN3-Q84 was induced in differentiated SH-SY5Y cells; cells were harvested 0, 3, 6 and 12 days post-induction (lanes 1 to 4) and their lysates analyzed by Western blotting to determine the levels of ATM-S1981, total ATM, γH2AX-S139, total H2AX, Chk2-T68, total Chk2, p53-S15, p53-S20, p53-S46 and total p53; β-actin was used as the loading control in A, C and E. **(B)** Levels of ATM-S1981, γH2AX-S139, Chk2-T68, p53-S15, p53-S20 and p53-S46 relative to their respective total protein levels in cells expressing ATXN3-Q84. Cells were harvested 0 (Grey), 3 (black), 6 (blue) and 12 (red) days post ATXN3-Q84 expression in Figs. B and D (n = 4, data represent mean ± SD, *** = p < 0.001 for B and F) **(C)** Expression of ATXN3-Q28 was induced in differentiated SH-SY5Y cells; cells were harvested 0, 3, 6 and 12 days post-induction (lanes 1 to 4) and their lysates analyzed by Western blotting to determine ATM-S1981, total ATM, γH2AX-S139, total H2AX, p53-S15 and total p53 levels. **(D)** Cells expressing ATXN3-Q28 were analyzed as in B and relative levels of ATM-S1981, γH2AX and p53-S15 vs. the respective total protein levels are shown; NS denotes non-significant. **(E)** Total protein was isolated from the deep cerebellar nuclei (DCN) of SCA3 transgenic mice (24 weeks old) constitutively expressing human mutant ATXN3 (lanes 3 and 4) and age-matched control mice (lanes 1 and 2) and analyzed by Western blotting to determine ATM-S1981, total ATM, γH2AX-S139, total H2AX, p53-S15 and total p53 levels. Each lane represents total protein from a pool of DCN tissue from 4–5 wild-type or an equal number of transgenic littermates. **(F)** Relative levels of ATM-S1981, γH2AX, p53-S15 with respect to total protein in SCA3 transgenic mouse DCN (black bars) vs. age-matched control DCN (grey bars); each bar represents a pool of DCN tissue collected from 4 to 5 littermate mice (either wild-type or transgenic). Data represent mean ± SD; *** = p < 0.001.

Activated ATM coordinates cell cycle progression with the damage-response checkpoints and DNA repair to preserve genomic integrity, via a well-orchestrated signaling network [23]. To investigate whether mutant ATXN3 activates ATM signaling in SCA3, we expressed ATXN3-Q84 in differentiated SH-SY5Y cells and assessed activation of the ATM pathway. Expression of ATXN3-Q84 strongly activated the ATM pathway, inducing the phosphorylation of ATM and H2AX and ATM’s downstream substrates Chk2 and p53 (Figs. 4A and 4B). By contrast, expression of wild-type ATXN3-Q28 did not activate the ATM pathway (Figs. 4C and 4D), suggesting that mutant ATXN3 strongly activates the DNA damage-response pathway and the polyQ sequence length is important for ATM pathway activation. Likewise, expression of the mutant ATXN3 carrying 72 and 80 poly-glutamines (ATXN3-Q72 and ATXN3-Q80) in SH-SY5Y cells also strongly activated the DNA damage-response ATM pathway (S8 Fig.). Furthermore, to test whether mutant ATXN3 activates p53 and Chk2 via activating ATM, we pre-treated the cells with ATM inhibitor Ku55933 and expressed ATXN3-Q84 and assessed the activation of DNA damage response pathway. Consistent with our hypothesis, ATXN3-Q84 expression failed to stimulate phosphorylation of Chk2 and p53 in the presence of the ATM inhibitor Ku55933 (S9 Fig.), substantiating our interpretation that mutant ATXN3 stimulates the DNA damage response p53 pathway via activating ATM. The dramatic increase in ATM, H2AX, Chk2 and p53 phosphorylation (Figs. 4A and S8) and formation of 53BP1 and γH2AX foci (Fig. 3) in response to mutant ATXN3 expression suggest that mutant ATXN3-induced genomic DNA strand breaks/damage is sufficient to activate the DNA damage- response pathway. Further, analysis of the tissue from the deep cerebellar nuclei (DCN) from SCA3 transgenic mice (CMVMJD135 mice) constitutively expressing mutant ATXN3 showed robust activation of the ATM pathway (increased phosphorylation of ATM, H2AX and p53) (Figs. 4E and 4F), suggesting that mutant ATXN3 strongly activates the DNA damage-response pathway *in vivo*. To further test whether inactivation of *PNKP* by mutant ATXN3 stimulates the ATM pathway, we examined *PNKP-siRNA*-treated differentiated SH-SY5Y cells for ATM pathway activation. Our data showed robust activation of the ATM and p53 pathways in cells transfected with *PNKP-siRNA*, but not in cells transfected with *control-siRNA* (S10 Fig.). To rule out the possibility that DNA damage and subsequent activation of the DNA damage response might be due in part to non-specific off-target toxic effects of the *PNKP-siRNA*, we used micro-RNA-adapted RNA interference (*shRNAmir*) to achieve more specific knockdown of PNKP in cells, and assessed activation of the DNA damage-response pathway in these cells. Similar to our previous observation described in S7 Fig., depletion of *PNKP* in SH-SY5Y cells with *PNKP-shRNAmir* constructs also resulted in increased genomic DNA damage (53BP1 and γH2AX foci formation; shown by arrows; S11 Fig.), and marked activation of the DNA damage-response ATM pathway (S12 Fig.). Moreover, recent studies have indicated that a mutant ATXN3-mediated increase in oxidative stress might be responsible for inducing DNA damage and SCA3 pathology [22]. Since oxidative stress alone can activate the ATM pathway [24], we sought to determine whether mutant ATXN3 activates ATM via an oxidation-dependent mechanism. To test this possibility, we induced ATXN3-Q84 expression in differentiated SH-SY5Y cells pre-treated with the antioxidant N-acetyl cysteine (NAC). However, pre-treating cells with NAC did not block mutant ATXN3-mediated activation of the DNA damage-response pathway (S13A and S13B Figs.). Likewise, expression of ATXN3-Q84 strongly activated the ATM pathway in cells overexpressing the antioxidant enzyme catalase (S13C and S13D Figs.), suggesting that the mutant ATXN3-induced DNA damage-response ATM pathway activation is oxidation-independent.

### Mutant ATXN3 activates the pro-apoptotic p53 pathway by activating ATM in SCA3

**Figure 5:**
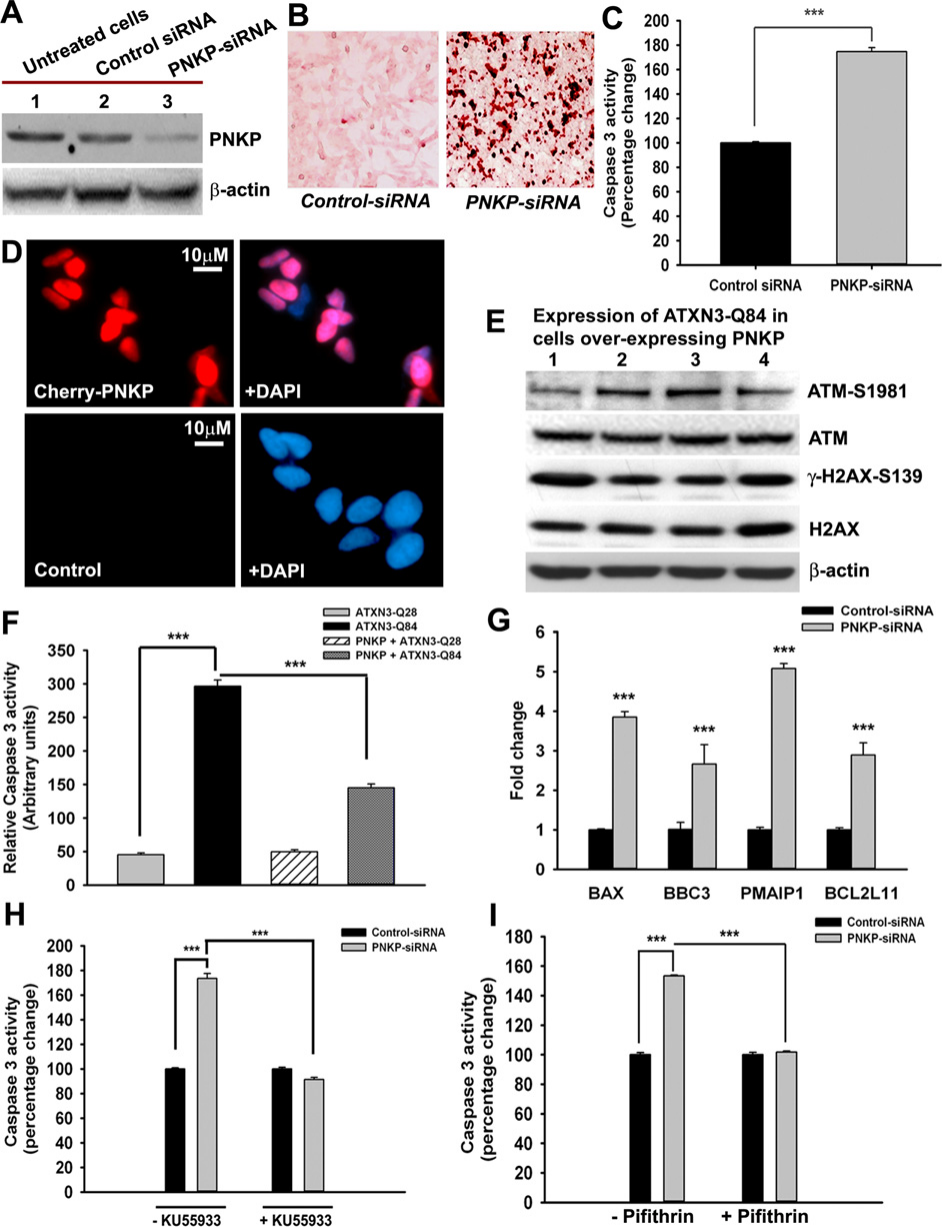
The mutant ATXN3-induced apoptotic pathway is rescued by PNKP-overexpression or inhibition of ATM and p53. **(A)** Total protein was isolated from control untreated SH-SY5Y cells (lane 1), SH-SY5Y cells transfected with *control-siRNA* (lane 2) and SH-SY5Y cells transfected with *PNKP-siRNA* (lane 3), the cells harvested 48 hours after transfection, and their lysates analyzed by Western blotting to determine the PNKP levels; β-actin was used as the loading control. **(B)** Light microscopic images showing TUNEL staining of SH-SY5Y cells transfected with *control-siRNA* or *PNKP-siRNA*. TUNEL staining was performed 48 hours post-transfection. **(C)** Caspase-3 activities in SH-SY5Y cells treated with *control-siRNA* or *PNKP-siRNA* were measured 48 hours after transfecting the cells with the respective siRNA. Data represent means ± SD; (n = 3, *** = p < 0.001) in C, and F-I. **(D)** Fluorescence microscopic images showing SH-SY5Y cells overexpressing Cherry-tagged PNKP and control untreated SH-SY5Y cells. **(E)** Expression of ATXN3-Q84 was induced in SH-SY5Y cells overexpressing PNKP, the cells harvested 0, 3, 6 and 12 days post-induction (lanes 1 to 4), and their lysates analyzed by Western blotting to determine ATM-S1981, total ATM, γ-H2AX-S139 and total H2AX levels; β-actin was used as the loading control. **(F)** Caspase-3 activities in cells expressing ATXN3-Q28 and ATXN3-Q84, co-expressing exogenous PNKP and ATXN3-Q28, and co-expressing PNKP and ATXN3-Q84. **(G)** SH-SY5Y cells were transfected with *PNKP-* or *control-siRNA;* 48 hours post-transfection, total RNA was isolated, and the expression of *BAX*, *BBC3, Bcl2L11* and *PMAIP1* determined by qRT-PCR. **(H)** Caspase-3 activities in SH-SY5Y cells transfected with *PNKP-siRNA, control-siRNA*; and in SH-SY5Y cells pre-treated with the ATM inhibitor Ku55933 and transfected with *control-siRNA* and *PNKP-siRNA*. Caspase-3 activities are expressed as percentage change normalized to the control in H and I. **(I)** Caspase-3 activities in SH-SY5Y cells transfected with *control-siRNA* and *PNKP-siRNA*, and in SH-SY5Y cells pre-treated with the p53 inhibitor Pifithrin-α and transfected with *control-siRNA* and *PNKP-siRNA*.

The transcription factor p53 is the primary target molecule in the ATM pathway, and many of the functions of ATM are p53-dependent. Activated p53 regulates a variety of cellular processes, such as transcription, cell cycle regulation, DNA damage-response repair and cell death [25–27]. The mutant polyQ proteins have been shown to induce p53-dependent apoptosis in SCA3, Huntington’s disease and spinocerebellar ataxia type 7 (SCA7) [15, 28, 29]. Ectopic expression of ATXN3-Q79 in cultured cerebellar neurons results in p53 activation, increased expression of *BAX*, increased release of cytochrome c from mitochondria, and apoptotic cell death [16]. However, the mechanism by which the polyQ proteins stimulate p53 activation and apoptosis remains unknown. We transfected the SH-SY5Y cells with either *PNKP-siRNA* or *control-siRNA* to test our hypothesis that the loss of PNKP activity triggers the pro-apoptotic signaling pathways in SCA3. Western blotting showed markedly decreased PNKP levels in cells treated with *PNKP-siRNA*, but not with *control-siRNA* (Fig. 5A). The TUNEL staining (Fig. 5B), and increased (~2-fold) caspase-3 activities (Fig. 5C) of the PNKP-depleted cells suggest robust activation of the pro-death pathways when PNKP was depleted. We thus developed SH-SY5Y cells overexpressing exogenous PNKP (Fig. 5D) and expressed the mutant ATXN3-Q84 in these cells to test whether PNKP overexpression blocks ATXN3-Q84-mediated cell death. The Western blot analysis clearly shows that ATXN3-Q84 failed to activate the ATM pathway in cells overexpressing PNKP (Fig. 5E). Moreover, overexpression of PNKP in these cells blocked ATXN3-Q84-mediated caspase-3 activation (Fig. 5F), suggesting that the loss of PNKP function plays an important role in mutant ATXN3-mediated cell death. Furthermore, quantitative RT-PCR (qRT-PCR) analysis of total RNA from the *PNKP-depleted* SH-SY5Y cells showed stimulated transcription of the p53-dependent pro-apoptotic genes such as *BAX*, *BBC3* (encoding PUMA), *Bcl2L11* (encoding BIM) and *PMAIP1* (encoding NOXA) (Fig. 5G). Moreover, we found that pre-treating the cells with either the ATM inhibitor Ku55933 or the p53 inhibitor Pifithrin-α could block *PNKP-siRNA*-induced caspase-3 activation (Figs. 5H and 5I). Likewise, expression of ATXN3-Q84 stimulated caspase-3 activity, whereas pre-treating the cells with Ku55933 or Pifithrin-α ameliorated the ATXN3-Q84-induced caspase-3 activation (S14A and S14B Figs.). These data substantiate our previous interpretation that mutant ATXN3 activates the p53-dependent pro-death pathway by activating ATM, and that chronic activation of the ATM→p53 pathway plays a pivotal role in mediating neuronal death in SCA3.

### Mutant ATXN3 facilitates nuclear translocation of protein kinase C δ (PKCδ) by activating the ATM pathway to amplify pro-apoptotic signals in SCA3

**Figure 6:**
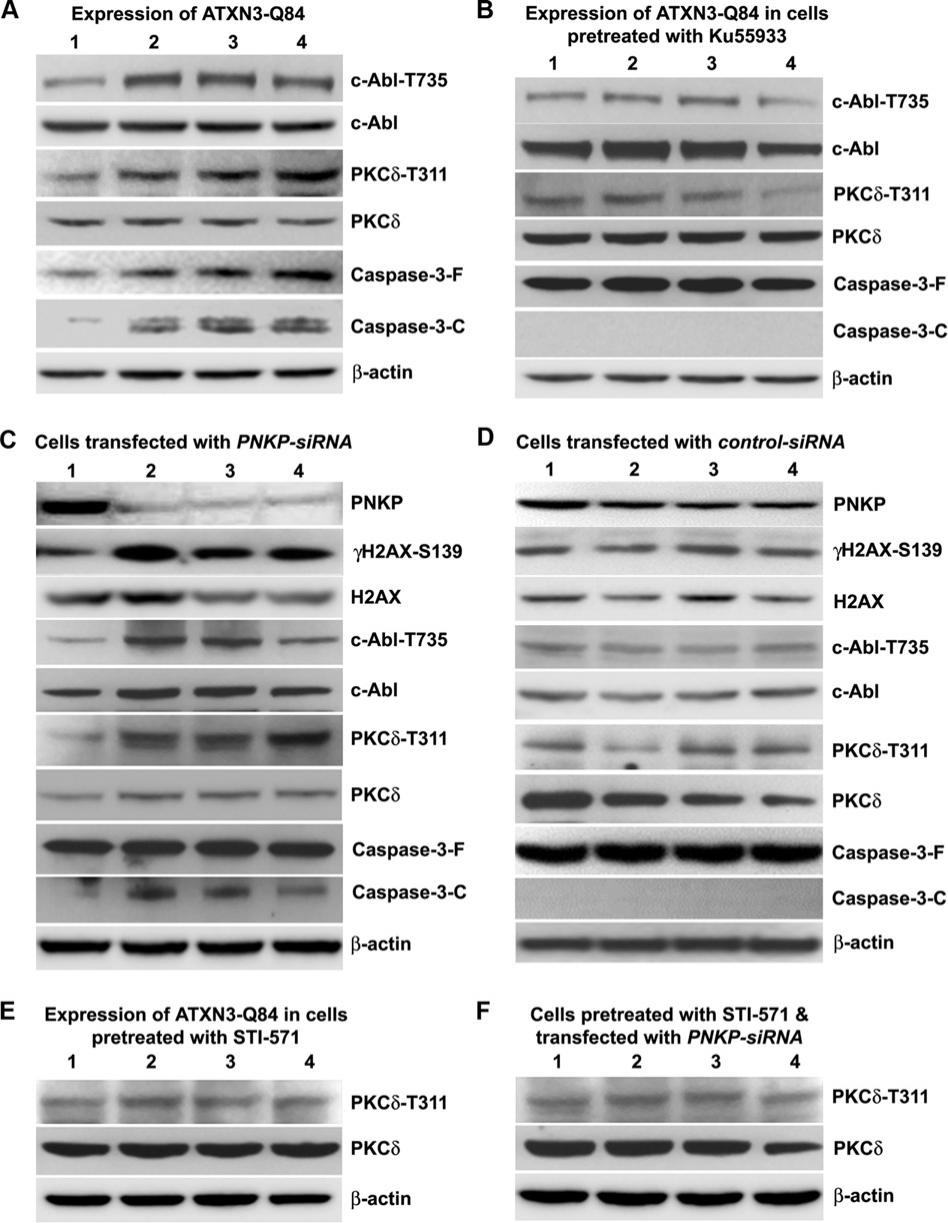
ATXN3-Q84 expression or PNKP deficiency activates the c-Abl→PKCδ signaling pathway. **(A)** SH-SY5Y cells were differentiated, the expression of ATXN3-Q84 was induced, and cells were harvested 0, 3, 6 and 12 days post-induction (lanes 1 to 4). Cell lysates were analyzed by Western blotting to determine the levels of c-Abl-T735, total c-Abl, PKCδ-T311, total PKCδ, full-length caspase-3 (-F), and cleaved caspase-3 (-C); β-actin was used as a loading control in A to F. **(B)** SH-SY5Y cells were differentiated, and treated with the ATM inhibitor Ku55933, expression of ATXN3-Q84 induced, and the cell lysates analyzed as in A. **(C)** SH-SY5Y cells were differentiated, transfected with 0, 50, 100 and 200 pmoles of *PNKP-siRNA* (lanes 1 to 4), harvested 48 hours post-transfection, and the cell lysates analyzed as in A. **(D)** SH-SY5Y cells were differentiated, transfected with 0, 50, 100 and 200 pmoles of *control-siRNA* (lanes 1 to 4), harvested 48 hours post-transfection, and the cell lysates analyzed as in C. **(E)** SH-SY5Y cells were differentiated, incubated with the c-Abl inhibitor STI-571, and expression of ATXN3-Q84 induced. The cells were harvested 0, 3, 6 and 12 days post-induction (lanes 1 to 4) and their lysates analyzed by Western blotting to determine PKCδ-T311 and total PKCδ levels. **(F)** SH-SY5Y cells were differentiated, and incubated with STI-571, and transfected with 0, 50, 100 and 200 pmoles (lanes 1 to 4) of *PNKP-siRNA*. Cells were harvested 48 hours post transfection and the cell lysates analyzed as in E.

In response to DNA damage, the tyrosine kinase c-Abl (encoded by the mammalian homolog of the *v-Abl* oncogene from the Abelson murine leukemia virus) is phosphorylated by ATM and DNA-dependent protein kinase, DNA-PK [30–32]. Activated c-Abl constitutively associates with PKCδ, resulting in the latter’s phosphorylation and nuclear translocation [33, 34]. Cytosolic retention of PKCδ is required to maintain cell survival, whereas its phosphorylation and nuclear translocation activates an apoptotic pathway [33–36]. Since ATXN3-Q84-induced DNA damage strongly activated the ATM pathway, we explored the possibility that ATXN3-Q84 could induce the phosphorylation of the ATM target proteins c-Abl and PKCδ. Expression of ATXN3-Q84 indeed increased the phosphorylation of c-Abl (T735) and PKCδ (T311), and caspase-3 cleavage (Figs. 6A and S15A). However, pre-treating the cells with Ku55933 blocked these events (Figs. 6B and S15B). Furthermore, consistent with our data in S10 Fig. showing marked activation of ATM pathway upon PNKP depletion, we found increased phosphorylation of c-Abl and PKCδ and higher caspase-3 activity in cells treated with *PNKP-siRNA* (Figs. 6C and S15C), but not with *control-siRNA* (Figs. 6D and S15D). However, depleting *PNKP* in cells pre-treated with Ku55933 stimulated the DNA damage response (assessed by increased H2AX phosphorylation), but did not increase the phosphorylation of c-Abl and PKCδ or caspase-3 cleavage (S16 Fig.). To test whether ATXN3-Q84 activates PKCδ by activating c-Abl, we pre-treated the cells with the c-Abl kinase inhibitor STI-571; ATXN3-Q84 expression failed to enhance PKCδ phosphorylation in cells pre-treated with STI-571 (Figs. 6E and S15E). Moreover, *PNKP* depletion did not enhance PKCδ phosphorylation when cells were pre-treated with STI-571 (Figs. 6F and S15F), suggesting that mutant ATXN3 increases PKCδ phosphorylation by activating the ATM→c-Abl signaling pathway.

**Figure 7:**
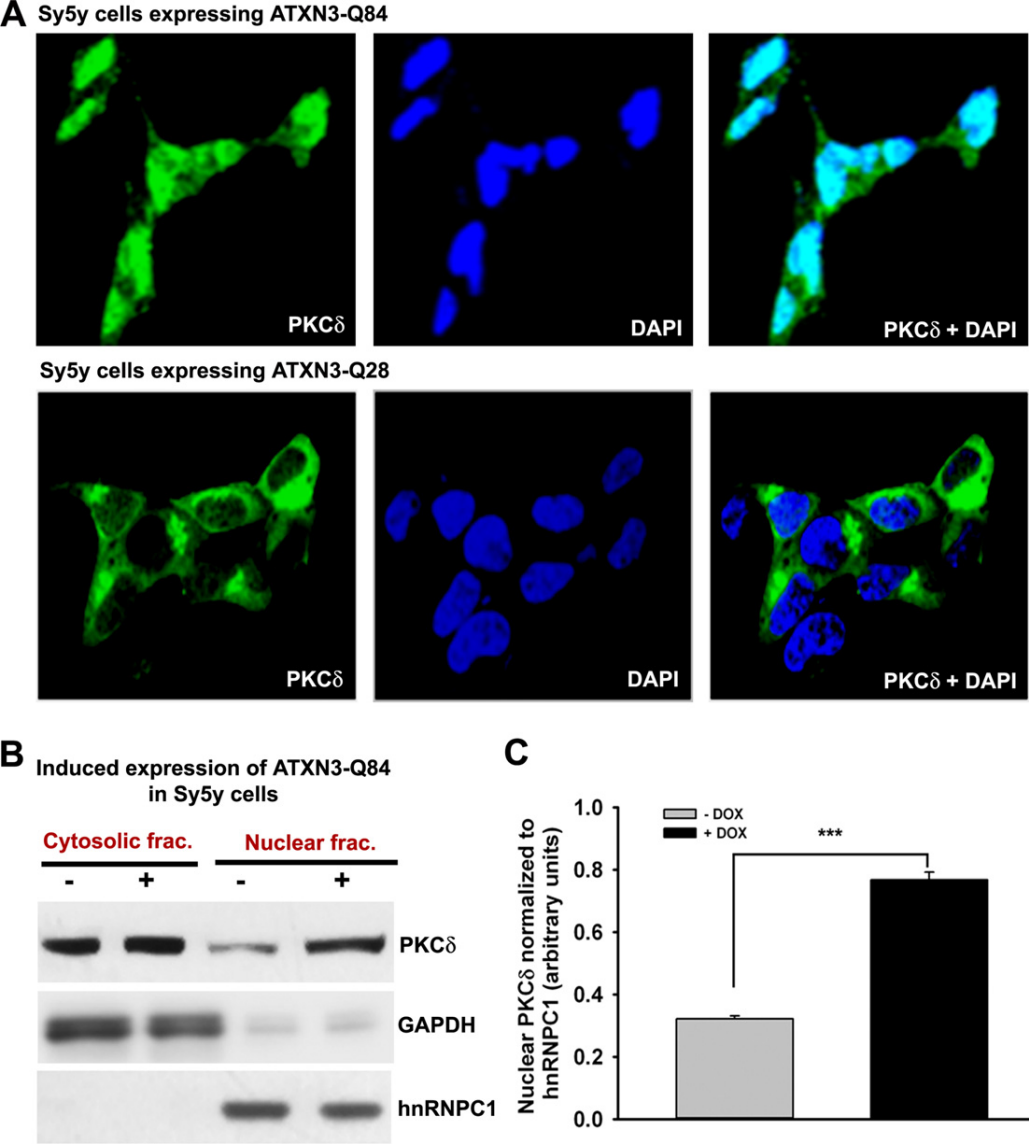
Expression of ATXN3-Q84 in SH-SY5Y cells facilitates nuclear translocation of PKCδ. **(A)** SH-SY5Y cells (2 × 10^4^ cells) were plated on chamber slides, expression of ATXN3-Q84 or ATXN3-Q28 was induced, and cells were analyzed by immunostaining with anti-PKCδ antibody (green). The subcellular distribution of PKCδ was assessed by confocal microscopy; nuclei were stained with DAPI. **(B)** Nuclear and cytosolic protein fractions were isolated from the SH-SY5Y cells expressing ATXN3-Q84 and control un-induced SH-SY5Y cells and the protein fractions analyzed by Western blotting with anti-PKCδ antibody to determine the relative nuclear vs. cytosolic abundance of PKCδ in induced (+) vs. uninduced (-) cells; GAPDH and hnRNPC1 were used as cytosolic and nuclear markers, respectively. **(C)** Relative levels of PKCδ in nuclear protein fractions of SH-SY5Y cells expressing ATXN3-Q84 (Dox-treated) vs. control cells (Dox-untreated). Data represent mean ± SD (n = 4; *** = p < 0.001).

Since phosphorylated PKCδ is translocated to nuclei [33–34] and we observed that ATXN3-Q84 stimulates PKCδ phosphorylation (Figs. 6 and S15), we assessed the relative abundance of PKCδ in the sub-cellular compartments of SH-SY5Y cells expressing ATXN3-Q84 and ATXN3-Q28. Cells expressing ATXN3-Q84 showed higher nuclear levels of PKCδ (Fig. 7A; upper panel); by contrast, cells expressing ATXN3-Q28 showed predominantly cytosolic PKCδ (Fig. 7A, lower panel). Western blotting of the nuclear and cytosolic protein fractions showed higher nuclear levels of PKCδ in ATXN3-Q84-expressing cells than in control cells (Figs. 7B and 7C). Depletion of *PNKP* also resulted in marked nuclear accumulation of PKCδ (S17 Fig.). Furthermore, immunohistological analysis revealed that about 60 to 70% of the nuclei had significant nuclear accumulation of PKCδ in the SCA3 transgenic mouse brain sections (S18 Fig. lower panels; arrows); in contrast, control mouse brains showed predominantly cytoplasmic PKCδ (S18 Fig. upper panels; arrowheads).

**Figure 8:**
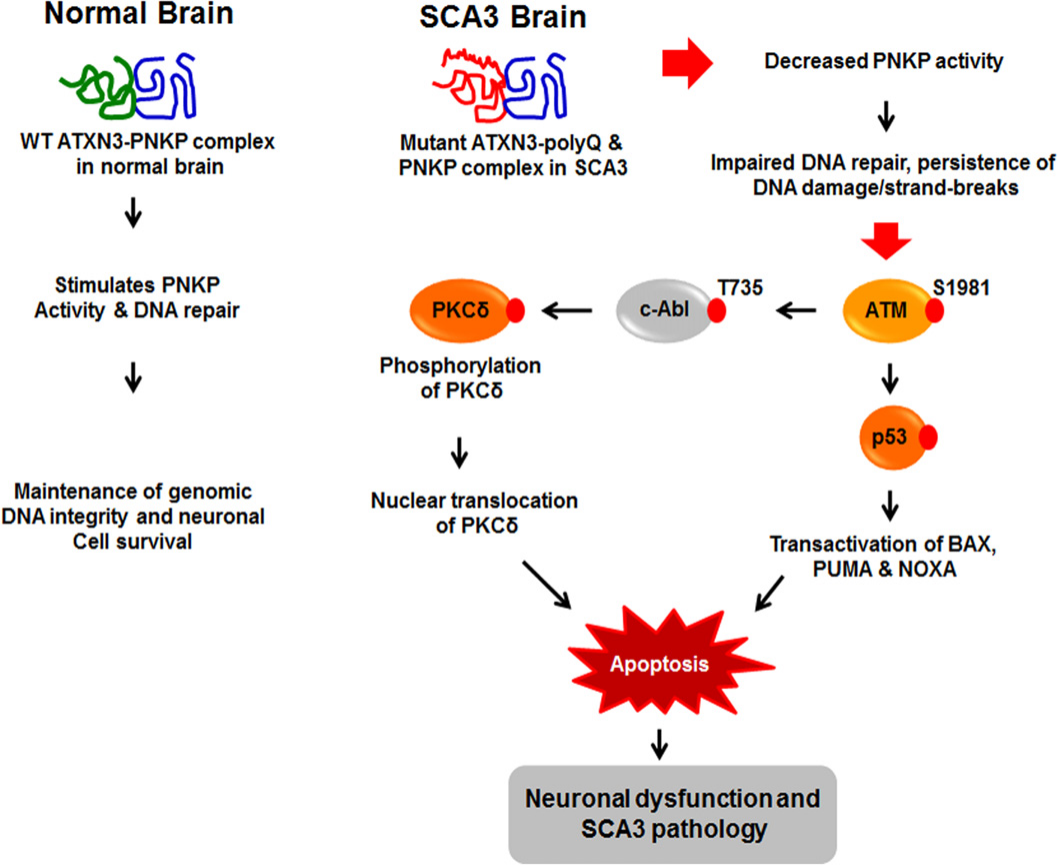
Proposed mechanism where interaction of PNKP with mutant expanded polyQ-containing ATXN3 abrogates PNKP’s 3’-phosphatase activity. This atypical interaction perturbs the efficacy of DNA repair, leading to the persistent accumulation of DNA damage/strand breaks in SCA3. The wild-type ATXN3 binds with and stimulates PNKP’s enzymatic activity to repair damaged DNA, contributing to genomic DNA sequence integrity and neuronal survival. By contrast, mutant ATXN3 interacts with and decreases PNKP’s 3’-phosphatase activity, leading to the persistent accumulation of DNA damage in the post-mitotic neurons and resulting in chronic activation of the DNA damage-response ATM→p53 signaling pathway in SCA3. This triggers apoptosis by increasing expression of such p53 target genes as BAX, PUMA and NOXA in SCA3. In parallel, activated ATM phosphorylates c-Abl kinase, which in turn phosphorylates PKCδ, facilitating nuclear translocation of PKCδ and thus further amplifying the pro-apoptotic output in SCA3 ultimately leading to neuronal death in SCA3.

We next assessed whether blocking PKCδ phosphorylation by inhibiting c-Abl kinase activity ameliorates mutant ATXN3-mediated caspase-3 activation. Pre-treating cells with STI-571 significantly inhibited mutant ATXN3-Q84-mediated caspase-3 activation (S19A Fig.). Likewise, pre-treating cells with STI-571 also ameliorated *PNKP-siRNA*-mediated caspase-3 activation (S19B Fig.). Collectively, these data, together with the data presented in the accompanying manuscript by Chatterjee et al, suggest that the wild-type ATXN3 interacts with and stimulates PNKP’s 3’-phosphatase activity, and this interaction possibly modulates the efficacy of DNA repair, and helps maintain genomic integrity and neuronal survival. In contrast, mutant ATXN3 interacts with PNKP and abrogates its 3’-phosphatase activity, resulting in increased accumulation of DNA damage that chronically activates ATM→p53 and ATM→c-Abl→PKCδ pro-apoptotic signaling to trigger neuronal dysfunction and apoptosis in SCA3 (illustrated in Fig. 8).

## Discussion

The DNA damage-response pathway is rapidly activated in response to DNA damage to repair the damaged sites by activating a well-orchestrated signaling network. However, if the DNA damage/lesions are irreparable, the damage-response pathway activates pro-death signaling cascades to ensure apoptotic demise of the damaged cells to maintain cell and tissue homeostasis. Due to their high metabolic activity, the post-mitotic neurons generate higher amounts of reactive oxygen species, and also have a higher risk of accumulating strand breaks due to their high transcriptional activity [37–41]. Therefore, post-mitotic neurons have an elaborate mechanism to repair DNA strand breaks/lesions to ensure longevity and functionality when subjected to insults [37, 38, 42, 43]. An emerging picture suggests that mutation or loss of function of DNA repair genes in post-mitotic neurons results in the accumulation of DNA damage/strand breaks, neuronal dysfunction and systemic neurodegeneration [44–49]. For example, mutations in the DNA repair enzymes *APTX* and *TDP1* have been shown to contribute to neurodegeneration and the development of ataxia in autosomal recessive disorders such as AOA1 (ataxia with oculomotor apraxia type 1) [44, 45] and SCAN1 (spinocerebellar ataxia with axonal neuropathy) respectively [46]. Another recent discovery suggests that mutations in FUS (fused in sarcoma), a DNA repair protein that associates with the HDAC1-SIRT1 repair complex, result in the accumulation of DNA strand breaks, neurodegeneration and neurological defects in amyotrophic lateral sclerosis (ALS) [47]. Moreover, using genome-wide linkage analysis in consanguineous families of an autosomal recessive disease, multiple point mutations were identified in *PNKP* that result in neurological phenotypes characterized by microcephaly, intractable seizures, and developmental delay [48]. The presence of a homozygous frame-shift mutation in *PNKP* was recently identified in an early-onset neurodegenerative disorder that manifests with polyneuropathy, cerebellar atrophy, microcephaly, epilepsy and intellectual disability [49]. Disease-causing point mutations ablate either the kinase or phosphatase activities of PNKP *in vitro* [50]. These findings explain how the loss of function of essential DNA repair enzymes and subsequent defective DNA repair and accumulation of DNA damage results in neurological abnormalities.

Recent studies also indicate that persistent accumulation of DNA damage and inappropriate activation of the DDR pathway may contribute to the pathogenic mechanism of fragile X mental retardation syndrome (Fragile X syndrome), a common form of inherited mental retardation caused by the loss of the fragile X mental retardation protein, FMRP [51, 52]. Accumulating evidence suggests that in addition to the translational regulation in the cytoplasm, FMRP is also present in the nuclei, where it strongly associates with the chromatin and plays an important role in the regulation of DDR pathway, maintenance of genomic DNA sequence integrity and neuronal survival [52, 53]. A FMRP mutant *Drosophila* has been shown to be hypersensitive to genotoxic stress, and fails to survive to adulthood when exposed to stress [51]. The FMRP mutant fly also shows the presence of DNA damage and activation of the p53-dependent apoptotic pathways after irradiation [51]. These findings suggest that FMRP plays an important role in the maintenance of genomic DNA sequence integrity and neuronal survival. It is likely that the loss of FMRP may impair the efficacy of DNA repair, resulting in the persistent accumulation of neuronal DNA damage and inappropriate activation of the DNA damage response p53-dependent pro-apoptotic pathways to elicit neuronal death. However, further molecular and interventional studies are required to identify the interacting protein partners of FMRP in the nuclei to establish the exact role of FMRP in DNA repair, regulation of DDR pathway and maintenance of genomic DNA sequence integrity and to determine whether the loss of FMRP function contributes to aberrant activation of the DDR pathway and neurodegeneration in fragile X syndrome.

In the present study, we show that PNKP is inactivated via its interaction with the mutant ATXN3, and also in part due to its recruitment into the insoluble polyQ aggregates in SCA3 brain. Our data, together with the data presented in the accompanying manuscript by Chatterjee et al., strongly support our interpretation that interaction of PNKP with mutant ATXN3 and/or trapping of PNKP in the polyQ aggregates markedly abrogate PNKP’s enzymatic activity and DNA repair efficacy, resulting in persistent accumulation of strand breaks in SCA3. Decreased PNKP activity and the presence of high levels of strand breaks in the SH-SY5Y cells expressing ATXN3-Q84, in SCA3 patients’ brain and SCA3 transgenic mouse brains expressing mutant ATXN3, strongly support this idea. Furthermore, we demonstrate that mutant ATNX3 potently activates the DNA damage-response ATM signaling pathway in SCA3, while increased phosphorylation of ATM, H2AX, Chk2 and p53, and the formation of 53BP1 and γH2AX foci in the ATXN3-Q84-expressing cells, SCA3 patients’ brains and SCA3 transgenic mouse brains suggest that mutant ATXN3 induces genomic DNA damage and chronically activates the ATM pathway in SCA3. Moreover, amelioration of ATXN3-Q84-mediated phosphorylation of p53, c-Abl and PKCδ by pharmacological inhibition of ATM suggests that mutant ATXN3 activates the pro-death signaling cascades via activating ATM in SCA3. The occurrence of higher levels of genomic DNA damage has been reported in several neurodegenerative diseases such as Huntington’s disease, Parkinson’s disease, Alzheimer’s disease, and ALS [54–58]. However, it remains to be determined whether higher genomic DNA damage and aberrant activation of the DNA damage-response pathway contributes to neurodegeneration and the etiology of these diseases.

Our data described in the present manuscript establish a mechanistic link between mutant ATXN3 expression and aberrant p53 pathway activation in SCA3, as previously reported [16, 22]. The transcription factor p53 regulates the cell cycle, DNA damage-response repair and cell death [25, 26, 59]. Activated p53 initiates neuronal death by activating the expression of *BAX, BBC3* and *PMAIP1* [26, 59–64], and these factors stimulate apoptosis by enhancing mitochondrial membrane permeabilization that facilitates the release of cytochrome c and Smac/DIABLO [65–67]. Mutant polyQ-containing proteins have been shown to activate p53-dependent apoptosis in SCA3, SCA7 and Huntington’s disease [15, 16, 28, 29]. Our data show elevated mRNA levels of *BAX*, *BBC3* and *PMAIP1* in ATXN3-Q84-expressing and *PNKP*-depleted cells. The amelioration of apoptosis by pharmacological inhibition of ATM and p53, suggest that mutant ATXN3-mediated aberrant activation of the DNA damage-response pathway facilitates the apoptotic demise of neuronal cells in SCA3. Moreover, in response to DNA damage, the protein tyrosine kinase c-Abl is phosphorylated by ATM and DNA-dependent protein kinase (DNA-PK) [30–32]. Activated c-Abl in turn phosphorylates and facilitates the nuclear translocation of PKCδ [33, 34], whose cytosolic retention is required to maintain cell survival, whereas phosphorylation and nuclear translocation of PKCδ activates the apoptotic pathway [34–36]. Phosphorylation of PKCδ allows its association with importin-α, resulting in nuclear translocation of PKCδ and activation of apoptosis [33–36]. Consistent with these reports, our data show that mutant ATXN3-mediated activation of ATM→c-Abl pathway enhanced the phosphorylation and facilitated the nuclear translocation of PKCδ in cells, and in SCA3 transgenic mouse brains. It has been suggested that nuclear PKCδ phosphorylates p73 and DNA-PK to facilitate apoptosis [68]; however, it is not yet clear how PKCδ triggers apoptosis. We are currently investigating whether mutant ATXN3 enhances the phosphorylation of p73 and DNA-PK to induce apoptosis in SCA3.

In conclusion, our current study provides compelling evidence of how mutant ATXN3 impedes the enzymatic activity of PNKP, and induces DNA damage that manifests with activation of the pro-apoptotic signaling pathways in SCA3. Our data suggest that in addition to activating the DNA damage-induced p53 pathway as described earlier in SCA3 [15, 16], mutant ATXN3 also activates the ATM-dependent cAbl→PKCδ pro-apoptotic pathway in parallel to cause neuronal dysfunction and eventually facilitating systemic neuronal degeneration in SCA3 brain (Fig. 8). Understanding the aberrant interaction between PNKP and mutant ATXN3 that results in the sustained activation of the pro-death signaling pathways may provide important insights to develop novel, mechanism-based therapeutic strategies for SCA3. Specific modulation of mutant ATXN3-mediated atypical activation of the DNA damage-response p53 and PKCδ pathways, or enhancing the efficacy of *in vivo* DNA damage repair may be effective strategies to combat the pathways leading to systemic neurodegeneration in SCA3. However, further investigation and interventional studies are required to device strategies to block this deleterious protein-protein interaction to rescue neuronal dysfunction and demise of neuronal cells in SCA3.

Finally, CAG repeat instabilities and expansions are causal factors for several other poly-Q expansion-related neurodegenerative diseases e.g., SCA1, SCA2, SCA6, SCA7, SCA17, DRPLA, SBMA and Huntington’s disease [2, 69]. Is has been challenging to understand why these repeats show a pronounced region-specific instability pattern in brain. However, a recent study has clearly shown significantly elevated expression levels of various DNA repair proteins in the cerebellum compared to the striatum, and consequent higher repeat instability in the striatum of HD mouse model [70]. This study suggests that lower activities of various DNA repair enzymes might be responsible for the higher instability of the CAG repeats observed in the striatum in HD brain compared to other regions of the brain. These data strongly suggest that optimal activities of various DNA repair proteins act as a safeguard against repeat instability and thus reduce somatic instability of the repeats in the specific brain region, dictating the severity of disease pathologies [70]. It will be interesting to determine whether the expression levels and/or activity of PNKP vary in various brain regions, which may provide important insight to understand the molecular basis of region-specific repeat instability and differential vulnerability of specific brain regions in different poly-glutamine expansion-associated neurological diseases.

## Materials and Methods

### Plasmid construction and generation of SH-SY5Y cell lines with inducible expression of ATXN3-Q28 and ATXN3-Q84

Plasmids pEGFP-Ataxin3-Q28, pEGFP-Ataxin3-Q84 and pFLAG-Ataxin3-Q80 were kindly provided by Dr. Henry L. Paulson (Addgene plasmids 22122, 22123 and 22129). Expression plasmid ATXN3-Q72 was kindly provided by Dr. Randall Pittman (University of Pennsylvania). The wild-type and mutant *ATXN3* cDNAs were sub-cloned in pAcGFPC1 (Clontech, USA) to construct plasmids pGFP-ATXN3-Q28, pGFP-ATXN3-Q84 and pGFP-ATXN3-Q80 respectively. The plasmids pGFP-ATXN3-Q28, pGFP-ATXN3-Q80 and pGFP-ATXN3-Q84 were digested with AgeI and MluI, and the GFP-ATXN3-Q28, GFP-ATXN3-Q80 and GFP-ATXN3-Q84 fragments were sub-cloned into the Tet-inducible plasmid pTRE-3G (Clontech, USA) using appropriate linkers. The trans-activator plasmid pTet-ON-3G (Clontech, USA) and responder plasmids pTRE-GFP-ATXN3-Q84, GFP-ATXN3-Q80 or pTRE-GFP-ATXN3-Q28 was co-transfected into SH-SY5Y cells, and positive clones were selected with G418. Stable SH-SY5Y clones inducibly expressing GFP-ATXN3-Q84, GFP-ATXN3-Q80 and GFP-ATXN3-Q28 were incubated with medium containing doxycycline (500 ng/ml), and expression of the transgene was assessed by Western blotting using anti-ATXN3 antibody. The *PNKP* cDNA was PCR-amplified from plasmid pWZL-Neo-Myr-Flag-PNKP (kindly provided by Drs. William Hahn and Jean Zhao; Addgene plasmid 20594) and sub-cloned into pcDNA3.1-Hygro (Invitrogen, USA) to construct plasmid pRP-PNKP. The catalase cDNA was PCR-amplified from a cDNA clone with appropriate primers and was sub-cloned into plasmid pcDNA3.1-Hygro to construct plasmid pRP-Catalase. Plasmid pRP-PNKP or pRP-Catalase was transfected into SH-SY5Y stable cell lines encoding inducible ATXN3-Q28 and ATXN3-Q84, and the clones were selected against hygromycin resistance. The effective expression of the exogenous PNKP and catalase in these cells were assessed by Western blot analyses with appropriate antibodies. Anti-PNKP mouse monoclonal antibody was a kind gift from Prof. Michael Weinfeld (University of Alberta, Canada) and anti-catalase antibody (Cat # SC-50508) was purchased from Santa Cruz Biotechnology, USA. The *PNKP* cDNA was PCR-amplified from plasmid pWZL-Neo-Myr-Flag-PNKP and the PCR product was sub-cloned into XhoI- and BamHI-digested pmCherryC1 (Clontech, USA) to construct pCherry-PNKP expressing Cherry-tagged PNKP.

### Cell culture and plasmid transfection

SH-SY5Y cells were purchased from ATCC and cultured in DMEM medium containing 15% FBS, and 1% B-27 supplement, and differentiated in DMEM medium containing 10% FBS, 1% B-27 supplement (Invitrogen, USA) and 20 μM retinoic acid. The SH-SY5Y stable cells encoding inducible GFPC1-ATXN3-Q28, GFPC1-ATXN3-Q72, GFPC1-ATXN3-Q80 and GFPC1-ATXN3-Q84 were differentiated for 7 days and transgene expression in the differentiated cells was induced by adding doxycycline to the medium to a final concentration of 500ng/ml. The *siRNA* duplexes for *PNKP* and control scrambled siRNA duplexes with same base composition were purchased from Sigma, USA, and transfection of the *PNKP-siRNA* duplexes into SH-SY5Y cells or differentiated SH-SY5Y cells were performed using *Lipofectamine RNAi-MAX* reagent (Invitrogen, USA). Plasmids encoding micro-RNA-based *PNKP-shRNAmir* and *control-shRNAmir* were purchased from Thermo Scientific, USA, and transfected into SH-SY5Y cells using Lipofectamine 2000 reagent (Invitrogen, USA).

### SCA3 autopsy brain tissue samples

Human autopsy specimens were obtained from SCA3 patients and control subjects in accordance with local legislation and ethical rules. Control brain samples were collected from age-matched individuals who were deemed free of neurodegenerative disorders. The SCA3 brain tissue samples used for this study were obtained from SCA3 patients who were clinically characterized by cerebellar ataxia, opthalmoplegia, dysarthria and dysphagia. The molecular diagnosis of SCA3 was established by analyzing genomic DNA extracted from peripheral blood using a combination of PCR and Southern blotting. The exact lengths of the expanded CAG repeat sequences in ATXN3 gene were established by sequencing the CAG repeat expansion loci of the mutant allele. All brain autopsies were frozen in liquid nitrogen immediately after surgery, and stored in a −80°C freezer until further analysis.

### SCA3 transgenic mice

The MJD transgenic mice used in this study were previously described [21]. CMVMJD135 mice express the human ATXN3 carrying 135 glutamines and develop a progressive neurological phenotype with onset at 6 weeks, which includes loss of strength, impairment of motor coordination, loss of balance and altered reflexes. At late stages they show an overall reduction in brain weight, with reduced volume and/or total cell number in pontine nuclei and deep cerebellar nuclei, and intranuclear ATXN3 inclusions in several disease-relevant regions of the brain and spinal cord. Transgenic mice and control non-transgenic littermate mice (n = 4–5 pools of two animals per genotype) with a mean age of 20 weeks were sacrificed by decapitation, and brain slices were obtained for the macrodissection of the deep cerebellar nuclei using a stereomicroscope (Model SZX7, Olympus America Inc., USA) and frozen at −80ºC. For immunofluorescence assays, transgenic and wild-type littermate mice (mean age: 24 weeks) were deeply anesthetized and transcardially perfused with sterile PBS followed by 4% paraformaldehyde (PFA) in PBS. Brains were post-fixed overnight in fixative solution and embedded in paraffin. Slides with 4-μm-thick paraffin sections were processed for immunostaining with anti-PKCδ, -ATXN3 and—PNKP antibodies.

### Ethics statement for animal usage

The SCA3 transgenic mice (CMVMJD135 transgenic mice) were sacrificed and the brain tissues were collected according to the standard approved procedure and national and international guidelines and animal protocols were strictly followed.

### Alkaline comet assays

Alkaline comet assays were performed using a Comet Assay Kit (Trevigen, USA). The brain tissue was homogenized in PBS at 4°C and sifted through a 300 μm sieve. Cells were suspended in 85 μL of ice-cold PBS and gently mixed with an equal volume of 1% low-melting agarose. The cell suspension was dropped onto an agarose layer, and incubated in lysis buffer for 1 hour. After lysis, slides were incubated in buffer containing 0.3 M NaOH, 1 mM EDTA (pH 13) for 40 min, and electrophoresed for 1 hour. After neutralization, slides were stained and analyzed with a fluorescent microscope. To assess genomic DNA damage after treatment with genotoxic agents, cells were treated with 10 μM hydrogen peroxide (H_2_O_2_) for 20 minutes in serum-free medium, washed twice with ice-cold PBS and subjected to comet analysis as described above.

### Antibodies and western blot analysis

The antibodies for p53 (Cat # 9282), P-p53-S15 (Cat # 9286), P-p53-S20 (Cat # 9287), P-p53-S46 (Cat # 2521), Chk2 (Cat # 2662), P-Chk2-T68 (Cat # 2661), c-Abl (Cat # 2862), P-cAbl-T735 (Cat # 2864), PKCδ (Cat # 2058), P-PKCδ-T311 (Cat # 2055), p-53BP1-S1778 (CAT # 2675) and caspase-3 (Cat # 9668) were from Cell Signaling, USA; anti-H2AX (Cat # ab11175) and P-γH2AX-S139 (Cat # ab11174) were from Abcam, USA; anti-ATM (Cat # 1549–1) and P-ATM-S1981 (Cat # 2152–1) were from Epitomics, USA; anti-ataxin-3 rabbit polyclonal antibody (cat # 13505–1-AP) was from Protein tech, USA; anti-ataxin-3 mouse monoclonal antibody (Cat # Mab5360) was from Millipore, USA; and anti-polyQ diseases marker antibody, 5TF1–1C2 (cat # Mab1574) was from Millipore, USA. Cell pellets or mouse brain tissues were homogenized and total protein was isolated using a total protein extraction kit (Millipore, USA). The cytosolic and nuclear fractions were isolated from SH-SY5Y cells expressing ATXN3-Q84 and ATXN3-Q28 using a NE-PER nuclear protein extraction kit (Thermo Scientific, USA). The Western blot analyses were performed according to the standard procedure and each experiment was performed a minimum of 3 times to ensure reproducible and statistically significant results.

### Bimolecular fluorescence complementation (Bi-FC) assay

Bi-molecular fluorescence complementation assays were performed as described previously by Shyu et al [19]. Plasmids pBiFC-VN173 (encoding 1 to 172 N-terminal amino acids of modified GFP) and pBIFC-VC155 (encoding 155 to 238 C-terminal amino acids of modified GFP) were kindly provided by Dr. Chang-Deng Hu, Purdue University (Addgene plasmids 22011 and 22010). The ATXN3 cDNA (encoding 28 and 84 glutamines) were cloned in-frame with the C-terminal amino acids of modified GFP in plasmid pBiFC-VC155 and the *PNKP* cDNA was cloned in-frame with the N-terminal amino acids of modified GFP in plasmid pBIFC-VN173. SH-SY5Y cells (2×10^4^ cells) were grown on chamber slides and transfected after 24 hours of plating. Plasmids pVN173-PNKP and pVC155-ATXN3-Q28 or pVN173-PNKP and pVC155-ATXN3-Q84 were co-transfected into SH-SY5Y cells and reconstitution of green/yellow fluorescence was monitored by fluorescence microscopy to assess bimolecular protein-protein interactions. Transfections of pVC155-ATXN3-Q28, pVC155-ATXN3-Q84 and pVN173-PNKP as single plasmids into SH-SY5Y cells were used as negative controls.

### Immunohistochemical (IHC) analysis and TUNEL staining

Expression of ATXN3-Q28 and ATXN3-Q84 in SH-SY5Y cells was induced by incubating the cells with doxycycline (500 ng/ml), and after 48 hours of incubation, the cells were fixed with 4% paraformaldehyde in PBS for 30 minutes. For the *PNKP* depletion experiment, SH-SY5Y cells were grown on cover slips and transfected with *PNKP-siRNA;* 48 hours after transfection, the cells were fixed with 4% paraformaldehyde for 30 minutes. The fixed cells were immunostained with anti-p-53BP1, anti-γH2AX or anti-PKCδ antibodies. Frozen brain sections from SCA3 patients and control subjects were fixed in 4% paraformaldehyde, washed with PBS, and immunostained with anti-p-53BP1 and γH2AX antibodies. Paraffin-embedded transgenic and control mouse brain sections were deparaffinized and rehydrated, fixed in 4% paraformaldehyde for 30 minutes, washed, and immunostained with anti-p-53BP1, anti-γH2AX, and PKCδ antibodies. Slides were washed according to our standard protocol and the nuclei stained with DAPI (Molecular Probe, USA) and photographed under a confocal microscope. TUNEL staining of the SH-SY5Y cells transfected with *PNKP-siRNA* and *control-siRNA* was performed using an *in situ* Apoptosis Detection Kit per the manufacturer’s protocol (Calbiochem, USA).

### Drug treatment

SH-SY5Y cells or differentiated SH-SY5Y cells were incubated with 5mM of N-acetyl cysteine (NAC), the ATM inhibitor Ku55933, p53 inhibitor Pifithrin-α or c-Abl kinase inhibitor STI-571 for 3 hours before the expression of ATXN3-Q28 and ATXN3-Q84. The ATM inhibitor Ku55933 was purchased from EMD Biosciences, USA and Pifithrin-α and STI-571 were purchased from Santa Cruz Biotechnology, USA. Drugs were added to the cell culture medium to a final concentration of 1 µM, 2 µM or 5 μM and incubated for 3 hours before transgene induction; fresh medium with drugs was replaced after 12 hours. Cells were harvested at various time points for Western blot analyses and for isolating total RNA and qRT-PCR analyses.

### Image collection

Images were collected using a Zeiss LSM-510 META confocal microscope with 40X or 60X 1.20 numerical aperture water immersion objective. The images were obtained using two different lines of excitation (488 and 543 nm) by sequential acquisition. After excitation with 488 nm laser line emission was measured with a 505–530 nm filter and after excitation with 543 laser line emission was measured with a 560–615 nm filter. All images were collected using 4-frame-Kallman-averaging with a pixel time of 1.26 μs, a pixel size of 110 nm and an optical slice of 1.0 μm. Z-stack acquisition was done at z-steps of 0.8 μm. All orthogonal views were done with LSM 510 software at the Optical Microscopy Core Laboratory of UTMB.

### Caspase-3 activity measurements

Caspase-3 activities were measured using a Caspase-3 activity assay kit (BD Biosciences, USA). The Caspase 3 assay kit is based on the hydrolysis of the substrate, acetyl-Asp-Glu-Val-Asp p-nitroanilide (Ac-DEVD-pNA) by caspase 3, resulting in the release of the p-nitroaniline (pNA) moiety from the substrate, and released p-Nitroaniline (pNA) is detected at 405 nm. Comparison of the absorbance of pNA from the sample with the control allowed determination of the fold increase in caspase-3 activity and the relative caspase-3 activities are expressed in arbitrary units. SH-SY5Y cells encoding ATXN3-Q28 and ATXN3-Q84 were cultured in DMEM (Invitrogen, USA) containing 15% FBS and were cultured on 96 well dishes in DMEM (Invitrogen, USA) containing 15% FBS. Expression of ATXN3-Q28 and ATXN3-Q84 were induced in presence or absence of the drugs Ku55933 (2 μM), Pifithrin-α (10 μM) or STI-571 (5 μM) with doxycycline (500 ng/ml). The cells were harvested 3 days after induction and caspase-3 activities were measured. For measuring caspase-3 activities in the *PNKP* and *control-siRNA*-treated cells, the SH-SY5Y cells were cultured in 96-well dishes and transfected with *PNKP-siRNA* or *control-siRNA* in the presence or absence of Ku55933 (2 μM), Pifithrin-α (10 μM) or STI-571 (5 μM). The cells were harvested 48 hours after transfection, and caspase-3 activities were measured.

### Gene expression analysis by real-time quantitative RT-PCR

Total RNA was extracted from SH-SY5Y cells expressing ATXN3-Q28, ATXN3-Q84, *PNKP-siRNA* and *control-siRNA* using an RNA extraction kit (Qiagen, USA) and purified using the TURBO DNA-free DNAse Kit (Ambion, USA). Brain tissue (deep cerebellar nuclei) from SCA3 transgenic mouse expressing ATXN3-Q135 was homogenized in trizol reagent (Invitrogen, USA) and RNA was isolated as above. 1μg of total RNA was reverse-transcribed using an RT-PCR kit (Clontech, USA). A cDNA aliquot from each reaction was quantified and 500ng of cDNA from each reaction was used for real-time qRT-PCR. The qRT-PCR reactions were repeated three times; primers used for the analyses (BAX: PPH00078B; BBC3: PPH02204C; PMAIP1: PPH02090F; BCL2L11: PPH00893) were purchased from Qiagen, USA, and tested for accuracy, specificity, efficiency and sensitivity by the manufacturer.

### *In situ* proximity ligation assay (PLA)

PLA assays were performed by the method we described previously [18]. In brief, SCA3 and normal brain sections were fixed with 4% paraformaldehyde, permeabilized with 0.2% Tween-20 and washed with 1X PBS. Brain sections were incubated with primary antibodies for PNKP (mouse monoclonal) and ATXN3 (anti-ATXN3; rabbit polyclonal) or PNKP (mouse monoclonal) and DNA ligase 3 (rabbit polyclonal). These samples were subjected to PLAs using the Duolink PLA kit from O-Link Biosciences (Uppsala, Sweden). The nuclei were counterstained with DAPI, and the PLA signals were visualized in a fluorescence microscope (Nikon) at 20× magnification.

### Statistical analysis

All tabulated data are expressed as mean ± SD, except where otherwise indicated. Differences between mean values of two groups were analyzed by Student’s *t* tests after checking for variance distribution via Levene’s test. We tested all data for normal distribution using the Kolmogorov-Smirnov test, followed by two-way ANOVA test to evaluate overall group differences. This was followed by Tukey’s post-hoc test to determine pair-wise significance if the ANOVA test indicated that a significant difference was present in the data set. In all cases, probability values of 0.05 or less were considered to be statistically significant.

## Supporting Information

S1 Fig PNKP interacts with wild-type and mutant ATXN3 in cultured cells. Plasmids pGFPC1 (empty control vector) and pCherry-PNKP (cherry-tagged PNKP) were co-transfected into SH-SY5Y cells and co-localization of PNKP and control GFP was assessed by confocal microscopy; nuclei are stained with DAPI. The merge of green and red fluorescence from GFP and Cherry-tagged PNKP, respectively appears as yellow/orange fluorescence. Nuclei were stained with DAPI
(TIF)

S2 Fig PNKP antibody specifically detects endogenous PNKP. **(A)** SH-SY5Y cells were treated with either control or PNKP-siRNA, harvested 48 hours post-transfection and the nuclear extract (NE, 25 µg) analyzed. The coomassie-stained gel showing equal loading of protein from control and PNKP-siRNA depleted cells; (B) Western blot showing PNKP levels in the cells treated with either control or PNKP-siRNA, GAPDH was used as loading control; (**C**) SH-SY5Y cells were treated with control- or PNKP siRNA for either 24 or 48 hours, immunostained with anti-PNKP antibody. The confocal image analysis showing the presence of PNKP (red) in control but significantly reduced in the PNKP-siRNA treated cells; Nuclei were stained with DAPI
(TIF)

S3 Fig PNKP co-localizes with ATXN3 in human brain sections. Normal control brain sections and SCA3 patients’ brain sections (expressing mutant ATXN3 encoding Q79 and Q84) were analyzed by co-immunostaining with anti-PNKP (red) and anti-ATXN3 (green) antibodies; the merge of red and green fluorescence from PNKP and ATXN3 appears as yellow/orange fluorescence. Nuclei were stained with DAPI.
(TIF)

S4 Fig PNKP co-localizes with ATXN3 in wild-type control and SCA3 transgenic mouse brain sections. SCA3 transgenic (CMVMJD135, lower panels) and control (upper panels) mouse brain sections were immunostained with anti-PNKP (red), and anti-ATXN3 (green) antibodies; the merge of red and green fluorescence appears as yellow/orange fluorescence. Nuclei were stained with DAPI.
(TIF)

S5 Fig SCA3 human brain sections show the occurrence of genomic DNA damage/strand breaks. Normal control human brain sections (**panels A and B**), and SCA3 patients’ brain sections expressing ATXN3-Q84 (**Panel C**), ATXN3-Q72 (**panel D**) and ATXN3-Q79 (**panel E**; mutant ATXN3 encoding 84, 72 and 79 glutamines respectively) were analyzed with anti-P-53BP1 antibody (red) to assess DNA strand breaks (as 53BP1 foci; shown by arrows). Nuclei were stained with DAPI. (F) Relative numbers of 53BP1 foci in control and SCA3 patients’ brain sections (n = 3, data represents mean ± SD, *** = p < 0.001).
(TIF)

S6 Fig Comet assays of neuronal cells from SCA3 transgenic mouse brain sections show genomic DNA damage. **(A)** Single-cell gel electrophoresis (comet assay; electrophoresed from left to right) of neuronal cells from control (left panel) and SCA3 transgenic (SCA3-TG) mouse brains (right panel); neuronal cells from deep cerebellar nuclei (DCN) of the CMVMJD135 SCA3 transgenic mouse brains but not control cells show the presence of genomic DNA damage/fragmentation that appears as comet tails (arrows). **(B)** Relative genomic DNA damage (expressed as comet tail moment) in control cells vs. SCA3-TG neuronal cells (n = 100, data represent mean ± SD; *** = p < 0.001). **(C)** Comet assay of control cells before and after treatment with 10µM of hydrogen peroxide for 20 minutes; genomic DNA damage/fragmentation appear as comet tails (shown by arrows). **(D)** Comet analysis of SCA3-TG neuronal cells before and after treatment with 10µM of hydrogen peroxide for 20 minutes; genomic DNA damage appear as comet tails (shown by arrows). **(E)** Relative genomic DNA damage/fragmentation in control cells and SCA3-TG neuronal cells before and after treatment with 10 µM of hydrogen peroxide. Data represents mean ± SD (n = 100)., *** = p < 0.001; significantly different from untreated wild type cells: # = p < 0.001; significantly different from untreated mutant cells: † = p < 0.001 significantly different from wild type cells upon hydrogen peroxide treatment.
(TIF)

S7 Fig Targeted depletion of PNKP in cells induces strand breaks and activates the DNA damage response. **(A)** Total protein from SH-SY5Y cells (lane 1), from SH-SY5Y cells treated with control siRNA (lane 2), and SH-SY5Y cells treated with *PNKP-siRNA* (lane 3) was isolated and analyzed by Western blotting to determine PNKP levels; β-actin was used as loading control. (**B**) Relative PNKP levels normalized to β-actin in control SH-SY5Y cells, SH-SY5Y cells treated with *control-siRNA* and in SH-SY5Y cells treated with *PNKP-siRNA*. **(C)** SH-SY5Y cells were transfected with *PNKP- or control-siRNA* and analyzed by immunostaining with anti-P-53BP1-S1778 antibody (red); 53BP1 foci are shown by arrows. **(D)** Relative number of 53BP1 foci in the SH-SY5Y cells transfected with *control-siRNA* or *PNKP-siRNA* (n = 100; data represents mean± SD, *** = p < 0.001). **(E)** SH-SY5Y cells were transfected with *PNKP-* or *control-siRNA*, and analyzed by immunostaining with anti-γH2AX-S139 antibody (red); γH2AX foci are shown by arrows. **(F)** Relative number of γH2AX foci in the SH-SY5Y cells transfected with *control-siRNA* or *PNKP-siRNA* (n = 100; data represents mean± SD, *** = p < 0.001).
(TIF)

S8 Fig Expression of mutant ATXN3 in cells activates DNA damage-response signaling. **(A)** Expression of ATXN3-Q72 was induced in SH-SY5Y cells and cells were harvested 0, 3, 6 and 12 days post-induction (lanes 1 to 4); cell lysates were analyzed by Western blotting to determine the levels of ATM-S1981, total ATM, γH2AX-S139, total H2AX, Chk2-T68, total Chk2, p53-S15 and total p53; β-actin was used as a loading control in A and C. **(B)** Relative levels of ATM-S1981, γH2AX-S139, Chk2-T68 and p53-S15 with respect to corresponding total protein in cells expressing ATXN3-Q72. Cells were harvested after 0 (grey), 3 (black), 6 (blue) and 12 (red) days of ATXN3-Q72 expression (n = 3, *** = p < 0.001 in B and D). **(C)** Expression of ATXN3-Q80 was induced in SH-SY5Y cells; cells were harvested 0, 3, 6 and 12 days post-induction (lanes 1 to 4) and cell lysates analyzed by Western blotting to determine the levels of ATM-S1981, total ATM, γH2AX-S139, total H2AX, Chk2-T68, total Chk2, p53-S15 and total p53. **(D)** Relative levels of ATM-S1981, γH2AX, Chk2-T68 and p53-S15 with respect to the corresponding total protein in cells expressing ATXN3-Q80, shown as described in B.
(TIF)

S9 Fig Expression of mutant ATXN3 activates DNA damage-response signaling by activating ATM. **(A)** SH-SY5Y cells were differentiated, incubated with Ku55933, and expression of ATXN3-Q84 was induced. Cells were harvested 0, 3, 6 and 12 days post-induction (lanes 1 to 4), and the cell lysates analyzed by Western blotting to detect ATM-S1981, total ATM, γH2AX-S139, total H2AX, Chk2-T68, total Chk2, p53-S15, total p53; β-actin was used as loading control. **(B)** Relative levels of ATM-S1981, γH2AX, Chk2-T68 and p53-S15 with respect to the corresponding total proteins in cells expressing ATXN3-Q84 and pre-treated with Ku55933. Cells were harvested after 0 (grey), 3 (black), 6 (blue) and 12 (red) days of ATXN3-Q84 expression (data represent mean ± SD, n = 3, *** = p < 0.001).
(TIF)

S10 Fig Depletion of PNKP in SH-SY5Y cells activates DNA damage-response signaling. **(A)** SH-SY5Y cells were differentiated and transfected with 0, 50, 100 and 200 pmoles (lanes 1 to 4) of *PNKP-siRNA*; 48 hours after transfection, cells were harvested and their lysates analyzed by Western blotting to detect ATM-S1981, total ATM, γH2AX-S139, total H2AX, p53-S15, p53-S20, p53-S46 and total p53. β-actin was used as a loading control in A and C. **(B)** Relative levels of ATM-S1981, γH2AX-S139, p53-S15, p53-S20, p53-S46 with respect to the corresponding total proteins in cells transfected with 0 (grey), 50 (black), 100 (blue) and 200 (red) pmoles of *PNKP-siRNA* (upper panel). PNKP level normalized to β-actin is shown in lower panel; n = 3, data represent mean ± SD, *** = p < 0.001 in B and D. **(C)** SH-SY5Y cells were differentiated and transfected with 0, 50, 100 and 200 pmoles (lanes 1 to 4) of *control-siRNA*; 48 hours after transfection, cells were harvested and the cell lysates analyzed by Western blotting as in A. **(D)** Relative levels of ATM-S1981, γH2AX-S139, p53-S15, p53-S20 with respect to the corresponding total protein in cells transfected with 0 (grey), 50 (black), 100 (blue) and 200 (red) pmoles of *PNKP-siRNA* (upper panel). PNKP levels normalized to β-actin are shown in the lower panel.
(TIF)

S11 Fig Depletion of PNKP in SH-SY5Y cells activates the DNA damage response. **(A)** SH-SY5Y cells were transfected with plasmids expressing *PNKP-shRNAmir or control-shRNAmir* and the transfected cells analyzed by immunostaining with anti-P-53BP1-S1778 antibody (red); 53BP1 foci are shown by arrows. **(B)** Relative number of 53BP1 foci in SH-SY5Y cells transfected with plasmids encoding *control-shRNAmir* (upper panel) vs. *PNKP-shRNAmir* (lower panel). The data represent mean ± SD, n = 100, ***p = 0.001 in B and D. **(C)** SH-SY5Y cells were transfected with plasmids expressing either *PNKP-shRNAmir* or *control-shRNAmir* and the cells analyzed by immunostaining with anti-γH2AX-S139 antibody (red); γH2AX foci are shown by arrows. **(D)** Relative number of P-γH2AX foci in SH-SY5Y cells transfected with plasmids expressing either *control-shRNAmir* or *PNKP-shRNAmir*.
(TIF)

S12 Fig Depletion of PNKP in cells activates the DNA damage-response pathway. **(A)** SH-SY5Y cells were transfected with 0, 2, 4 and 8 µg (lanes 1 to 4) of plasmid DNA expressing *PNKP-shRNAmir*; 48 hours post-transfection the cells were harvested and their lysates analyzed by Western blotting to determine PNKP, ATM-S1981, total ATM, γH2AX-S139, total H2AX, Chk2-T68 and total Chk2 levels; β-actin was used as loading control in A and C. **(B)** Relative levels of ATM-S1981, γH2AX-S139, Chk2-T68 with respect to the corresponding total protein in SH-SY5Y cells transfected with plasmid expressing *PNKP-shRNAmir*. Cells were transfected with 0 (grey bar), 2 (black bar), 4 (blue) and 8 µg (red) of plasmid expressing *PNKP-shRNAmir* (n = 3, data represent mean ± SD; *** = p < 0.001 in B and D). **(C)** SH-SY5Y cells were transfected with 0, 2, 4 and 8 µg (lanes 1 to 4) of plasmid DNA expressing *control-shRNAmir*; 48 hours post- transfections the cells were harvested and their lysates analyzed by Western blotting as in A. **(D)** Relative levels of ATM-S1981, γH2AX-S139, Chk2-T68 with respect to the corresponding total protein in SH-SY5Y cells transfected with plasmid expressing *control-shRNAmir*.
(TIF)

S13 Fig Mutant ATXN3 activates the DNA damage-response pathway in a redox-independent fashion. **(A)** SH-SY5Y cells were differentiated, incubated with N-acetyl cysteine, and the expression of ATXN3-Q84 induced. Cells were harvested 0, 3, 6 and 12 days after induction (lanes 1 to 4), and their lysates analyzed by Western blotting to detect ATM-S1981, total ATM, γH2AX-S139 and total H2AX levels; β-actin was used as a loading control in A and C. **(B)** Relative levels of ATM-S1981 and γH2AX-S139 with respect to the corresponding total protein determined in the Western blots described in A. Cells were harvested after 0 (grey), 3 (black), 6 (blue) and 12 (red) days of ATXN3-Q84 expression in B and D; n = 3; data represent mean ± SD, *** = p < 0.001 in B and D. **(C)** Expression of ATXN3-Q84 was induced in SH-SY5Y cells overexpressing the antioxidant enzyme catalase; cell lysates were analyzed as in A. **(D)** Relative levels of ATM-S1981 and P-γH2AX-S139 with respect to the corresponding total protein were determined in Western blots described in C.
(TIF)

S14 Fig Pharmacological inhibition of the ATM→p53 pathway ameliorates ATXN3-Q84-mediated caspase-3 activation. **(A)** Caspase-3 activities in SH-SY5Y cells expressing ATXN3-Q84 measured before and after treatment with ATM inhibitor Ku55933. **(B)** Caspase-3 activities in SH-SY5Y cells expressing ATXN3-Q28 and ATXN3-Q84 were measured before and after treatment with the p53 inhibitor Pifithrin-α. Caspase-3 activities are expressed as percentage change normalized to control, and data represent means ± SD; (n = 3; *** = p < 0.001 in A and B).
(TIF)

S15 Fig Mutant ATXN3 activates the c-Abl→PKCδ pathway. **(A)** Relative levels of c-Abl-T735, PKCδ-T311 and cleaved caspase-3 (caspase-3-C) with respect to the corresponding total protein in SH-SY5Y cells expressing ATXN3-Q84 (n = 3; data represents mean ± SD, *** = p < 0.001 in A to F). Cells were harvested after 0 (grey), 3 (black), 6 (blue) and 12 (red) days of ATXN3-Q84 expression in A and B. **(B)** Relative c-Abl-T735 and PKCδ-T311 levels in SH-SY5Y cells expressing ATXN3-Q84 and pre-treated with Ku55933. **(C)** Relative γH2AX-S139, c-Abl-T735, PKCδ-T311, and cleaved caspase-3 levels with respect to total protein in SH-SY5Y cells transfected with 0 (grey), 50 (black), 100 (blue) and 200 (red) pmoles of *PNKP-siRNA*. **(D)** Relative γH2AX-S139, c-Abl-T735, PKCδ-T311, and cleaved caspase-3 levels in SH-SY5Y cells transfected with 0 (grey), 50 (black), 100 (blue) and 200 (red) pmoles of *control-siRNA*. **(E)** Relative PKCδ-T311 with respect to total PKCδ in cells pre-treated with STI-571 and expressing ATXN3-Q84. Cells were harvested after 0 (grey), 3 (black), 6 (blue) and 12 (red) of expression. **(F)** Relative PKCδ-T311 with respect to total PKCδ in cells pre-treated with STI-571 and transfected with 0 (grey), 50 (black), 100 (blue) and 200 (red) pmoles of *PNKP-siRNA*.
(TIF)

S16 Fig Mutant ATXN3 activates the c-Abl→PKCδ pathway via activating ATM. **(A)** SH-SY5Y cells were differentiated, incubated with Ku55933, transfected with 0, 50, 100 and 200 pmoles of *PNKP-siRNA* (lanes 1 to 4), and cell lysates analyzed by Western blotting to determine PNKP, γH2AX-S139, total H2AX, c-Abl-T735, total c-Abl, PKCδ-T311, total PKCδ, Caspase-3 (cleaved), caspase-3 (full-length); β-actin was used as loading control. **(B)** Relative levels of γH2AX-S139, c-Abl-T735, PKCδ-T311, cleaved caspase-3 with respect to the corresponding total protein in cells pre-treated with Ku55399 and transfected with 0 (grey), 50 (black), 100 (blue) and 200 (red) pmoles of *PNKP-siRNA*. PNKP levels were normalized to β-actin. Data represents mean ± SD, n = 3, *** = p < 0.001.
(TIF)

S17 Fig Depletion of *PNKP* facilitates nuclear translocation of PKCδ. **(A)** SH-SY5Y cells were transfected with *PNKP-* or *control-siRNA* and the transfected cells were immunostained with anti-PKCδ antibody (green). The subcellular distribution of PKCδ was assessed by confocal microscopy; nuclei were stained with DAPI. **(B)** Nuclear and cytosolic protein fractions were isolated from SH-SY5Y cells transfected with *PNKP-* or *control-siRNA* and analyzed by Western blotting with anti-PKCδ antibody to determine the relative nuclear/cytosolic abundance of PKCδ; GAPDH and hnRNPC1 were used as cytosolic and nuclear markers, respectively. **(C)** Relative levels of PKCδ in cytosolic and nuclear protein fractions in cells transfected with *PNKP-siRNA* vs. *control-siRNA*. Data represents mean ± SD (n = 4; *** = p < 0.001)
(TIF)

S18 Fig SCA3 transgenic mouse brain sections show increased nuclear PKCδ levels. Brain sections from the SCA3 transgenic mouse expressing mutant ATXN3-Q135 (CMVMJD135 mice) and control mouse were analyzed by immunostaining with anti-PKCδ antibody (red) to assess the sub-cellular distribution of PKCδ. Cytosolic presence of PKCδ in the control mouse brain sections are shown by arrowhead, and nuclear PKCδ in the transgenic mouse brain sections is shown by arrows; nuclei were stained with DAPI
(TIF)

S19 Fig Mutant ATXN3 activates pro-apoptotic pathway by activating c-Abl kinase pathway. **(A)** Caspase-3 activities in SH-SY5Y cells expressing ATXN3-Q28 and ATXN3-Q84, and expressing ATXN3-Q28 and ATXN3-Q84 in the presence of STI-571; caspase-3 activities are expressed as percentage change normalized to control. Data represent means ± SD; (n = 3; *** = p < 0.001). **(B)** Caspase-3 activities in SH-SY5Y cells transfected with *PNKP-* or *control-siRNA*, and in SH-SY5Y cells pre-treated with the c-Abl inhibitor STI-571 and transfected with *PNKP-siRNA*; caspase-3 activities are expressed as percentage change normalized to control. Data represent means ± SD (n = 3; *** = p < 0.001).
(TIF)
