# Supplementary figures and images for: Inactivation of PNKP by Mutant ATXN3 Triggers Apoptosis by Activating the DNA Damage-Response Pathway in SCA3
Source: PLoS Genet. 2015 Jan 15;11(1):e1004834. doi: 10.1371/journal.pgen.1004834 (PMC4295939; doi:10.1371/journal.pgen.1004834)

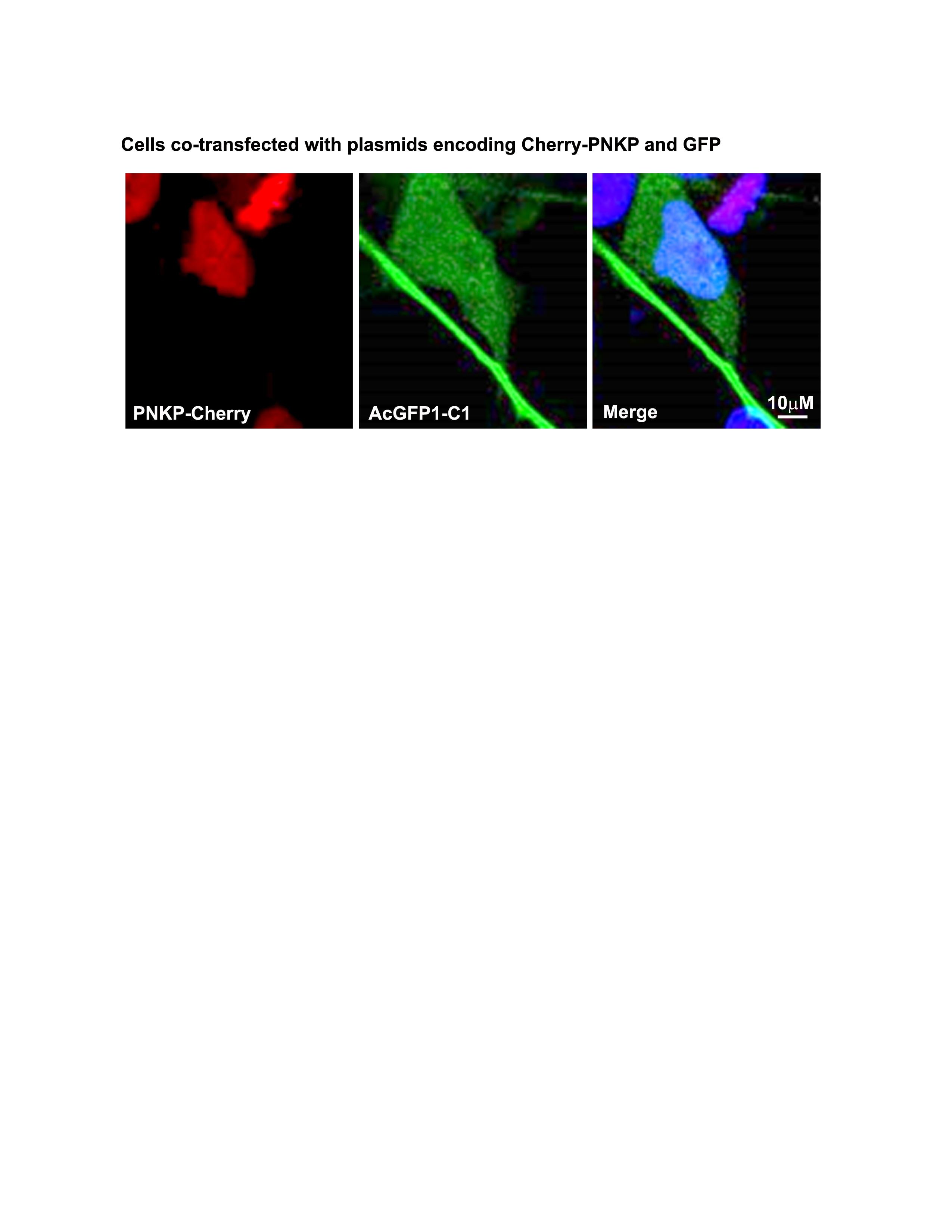

Supplement: S1 Fig — Plasmids pGFPC1 (empty control vector) and pCherry-PNKP (cherry-tagged PNKP) were co-transfected into SH-SY5Y cells and co-localization of PNKP and control GFP was assessed by confocal microscopy; nuclei are stained with DAPI. The merge of green and red fluorescence from GFP and Cherry-tagged PNKP, respectively appears as yellow/orange fluorescence. Nuclei were stained with DAPI (TIF) [file pgen.1004834.s001.tif]

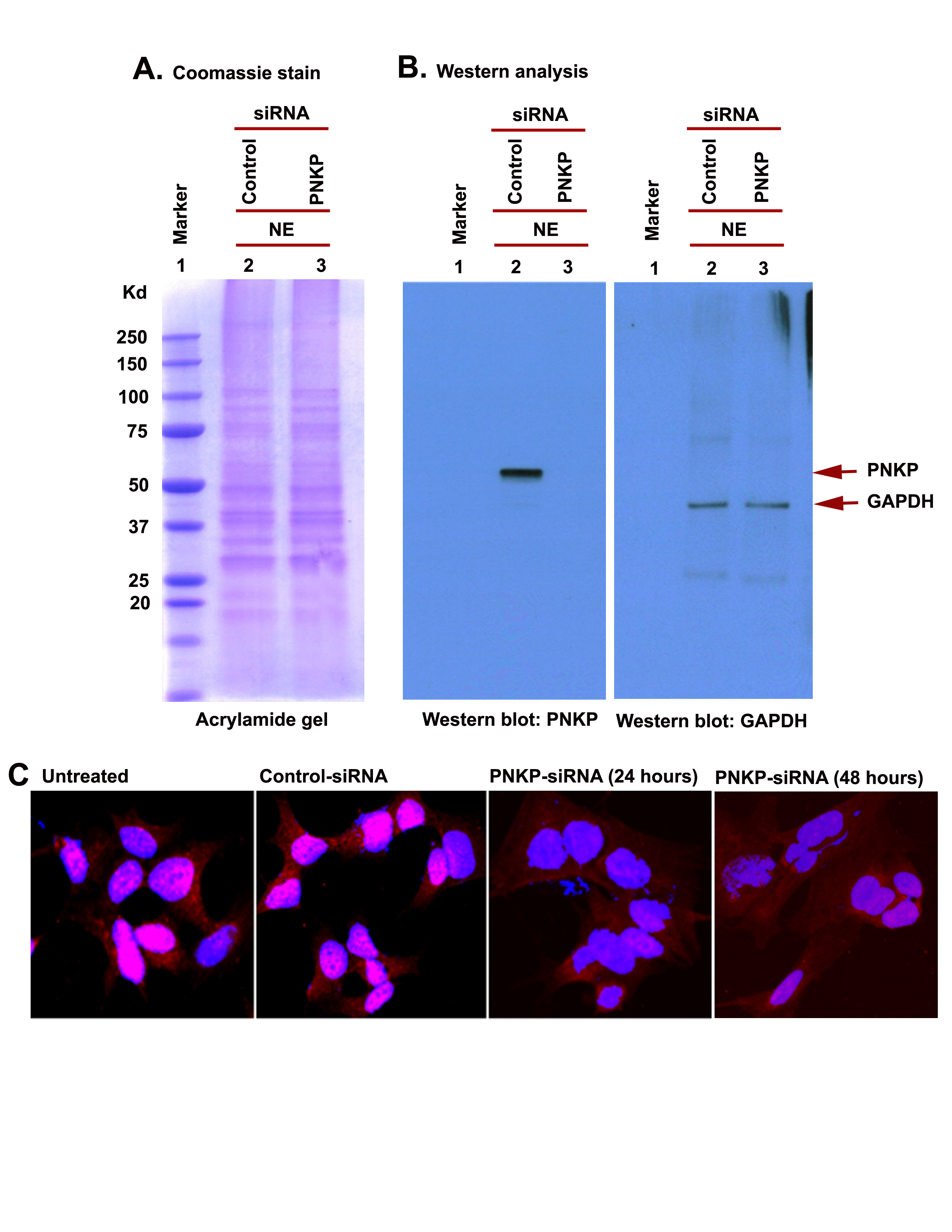

Supplement: S2 Fig — (A) SH-SY5Y cells were treated with either control or PNKP-siRNA, harvested 48 hours post-transfection and the nuclear extract (NE, 25 µg) analyzed. The coomassie-stained gel showing equal loading of protein from control and PNKP-siRNA depleted cells; (B) Western blot showing PNKP levels in the cells treated with either control or PNKP-siRNA, GAPDH was used as loading control; (C) SH-SY5Y cells were treated with control- or PNKP siRNA for either 24 or 48 hours, immunostained with anti-PNKP antibody. The confocal image analysis showing the presence of PNKP (red) in control but significantly reduced in the PNKP-siRNA treated cells; Nuclei were stained with DAPI (TIF) [file pgen.1004834.s002.tif]

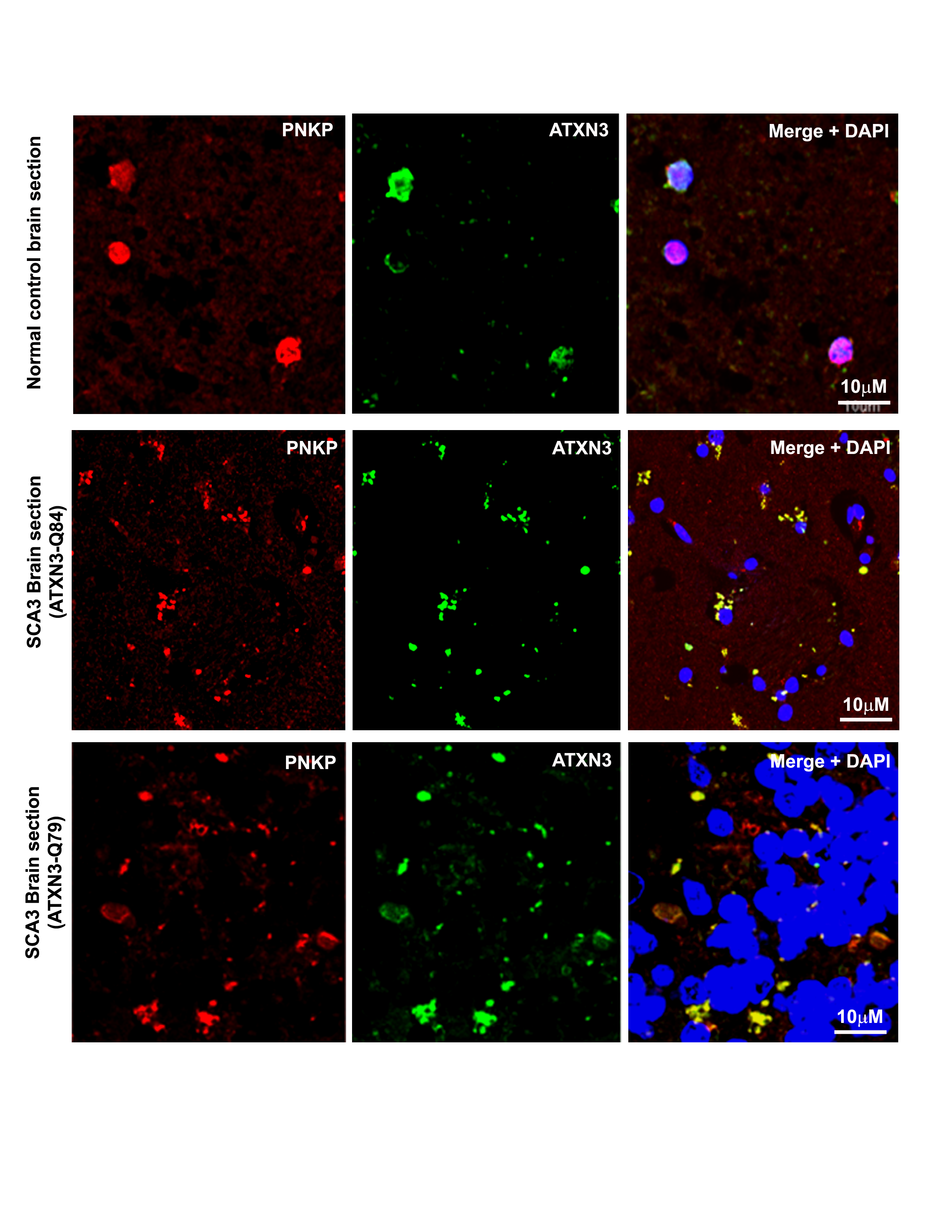

Supplement: S3 Fig — Normal control brain sections and SCA3 patients’ brain sections (expressing mutant ATXN3 encoding Q79 and Q84) were analyzed by co-immunostaining with anti-PNKP (red) and anti-ATXN3 (green) antibodies; the merge of red and green fluorescence from PNKP and ATXN3 appears as yellow/orange fluorescence. Nuclei were stained with DAPI. (TIF) [file pgen.1004834.s003.tif]

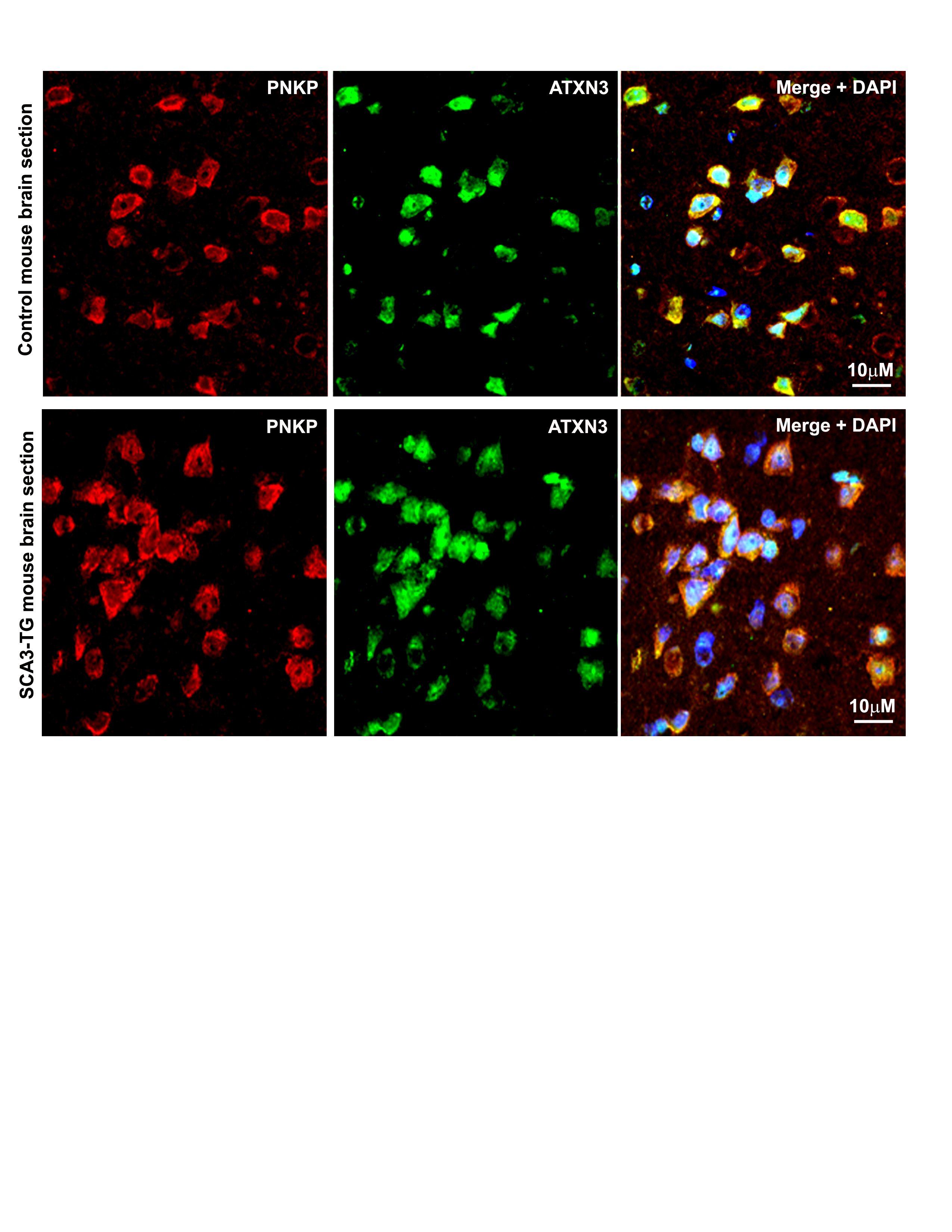

Supplement: S4 Fig — SCA3 transgenic (CMVMJD135, lower panels) and control (upper panels) mouse brain sections were immunostained with anti-PNKP (red), and anti-ATXN3 (green) antibodies; the merge of red and green fluorescence appears as yellow/orange fluorescence. Nuclei were stained with DAPI. (TIF) [file pgen.1004834.s004.tif]

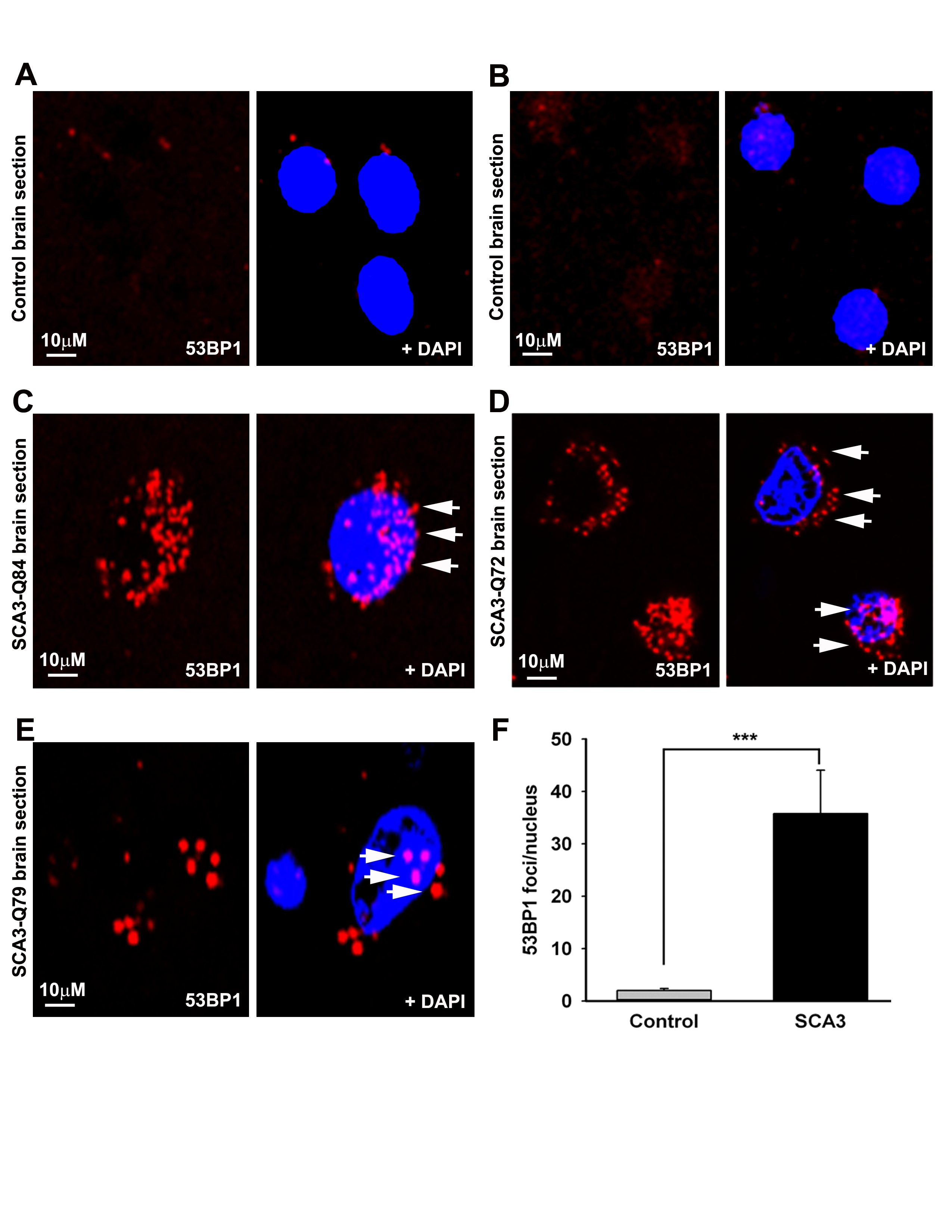

Supplement: S5 Fig — Normal control human brain sections (panels A and B), and SCA3 patients’ brain sections expressing ATXN3-Q84 (Panel C), ATXN3-Q72 (panel D) and ATXN3-Q79 (panel E; mutant ATXN3 encoding 84, 72 and 79 glutamines respectively) were analyzed with anti-P-53BP1 antibody (red) to assess DNA strand breaks (as 53BP1 foci; shown by arrows). Nuclei were stained with DAPI. (F) Relative numbers of 53BP1 foci in control and SCA3 patients’ brain sections (n = 3, data represents mean ± SD, *** = p < 0.001). (TIF) [file pgen.1004834.s005.tif]

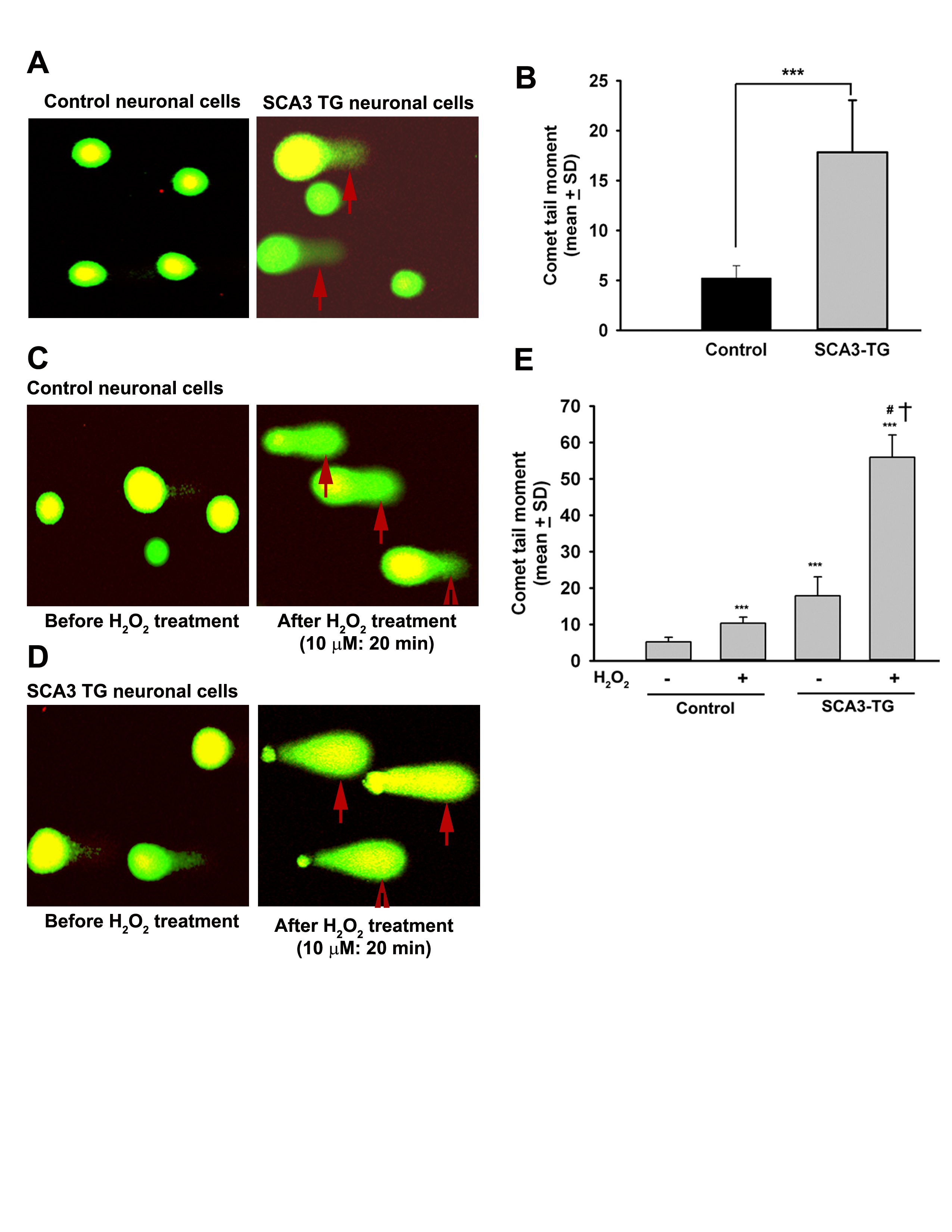

Supplement: S6 Fig — (A) Single-cell gel electrophoresis (comet assay; electrophoresed from left to right) of neuronal cells from control (left panel) and SCA3 transgenic (SCA3-TG) mouse brains (right panel); neuronal cells from deep cerebellar nuclei (DCN) of the CMVMJD135 SCA3 transgenic mouse brains but not control cells show the presence of genomic DNA damage/fragmentation that appears as comet tails (arrows). (B) Relative genomic DNA damage (expressed as comet tail moment) in control cells vs. SCA3-TG neuronal cells (n = 100, data represent mean ± SD; *** = p < 0.001). (C) Comet assay of control cells before and after treatment with 10µM of hydrogen peroxide for 20 minutes; genomic DNA damage/fragmentation appear as comet tails (shown by arrows). (D) Comet analysis of SCA3-TG neuronal cells before and after treatment with 10µM of hydrogen peroxide for 20 minutes; genomic DNA damage appear as comet tails (shown by arrows). (E) Relative genomic DNA damage/fragmentation in control cells and SCA3-TG neuronal cells before and after treatment with 10 µM of hydrogen peroxide. Data represents mean ± SD (n = 100)., *** = p < 0.001; significantly different from untreated wild type cells: # = p < 0.001; significantly different from untreated mutant cells: † = p < 0.001 significantly different from wild type cells upon hydrogen peroxide treatment. (TIF) [file pgen.1004834.s006.tif]

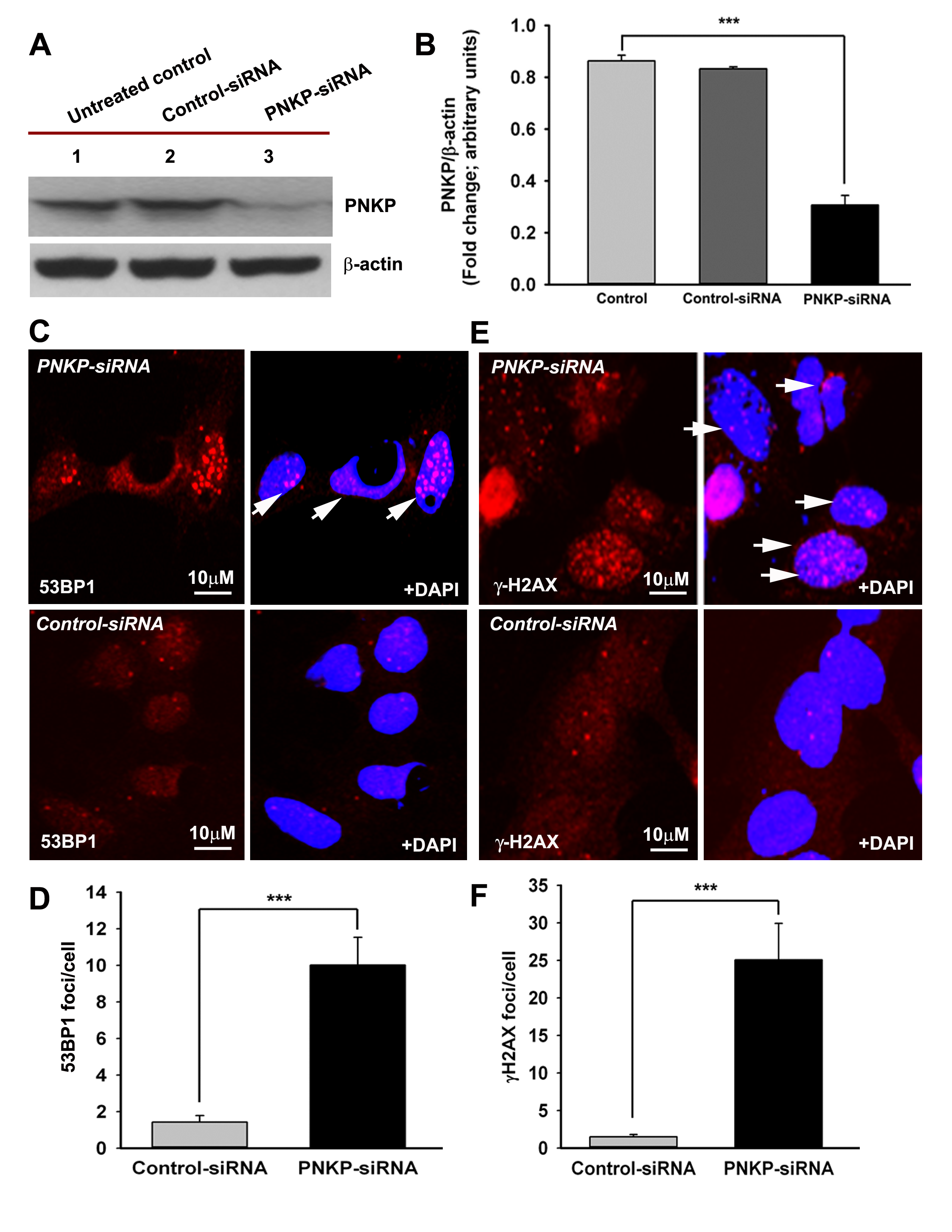

Supplement: S7 Fig — (A) Total protein from SH-SY5Y cells (lane 1), from SH-SY5Y cells treated with control siRNA (lane 2), and SH-SY5Y cells treated with PNKP-siRNA (lane 3) was isolated and analyzed by Western blotting to determine PNKP levels; β-actin was used as loading control. (B) Relative PNKP levels normalized to β-actin in control SH-SY5Y cells, SH-SY5Y cells treated with control-siRNA and in SH-SY5Y cells treated with PNKP-siRNA. (C) SH-SY5Y cells were transfected with PNKP- or control-siRNA and analyzed by immunostaining with anti-P-53BP1-S1778 antibody (red); 53BP1 foci are shown by arrows. (D) Relative number of 53BP1 foci in the SH-SY5Y cells transfected with control-siRNA or PNKP-siRNA (n = 100; data represents mean± SD, *** = p < 0.001). (E) SH-SY5Y cells were transfected with PNKP- or control-siRNA, and analyzed by immunostaining with anti-γH2AX-S139 antibody (red); γH2AX foci are shown by arrows. (F) Relative number of γH2AX foci in the SH-SY5Y cells transfected with control-siRNA or PNKP-siRNA (n = 100; data represents mean± SD, *** = p < 0.001). (TIF) [file pgen.1004834.s007.tif]

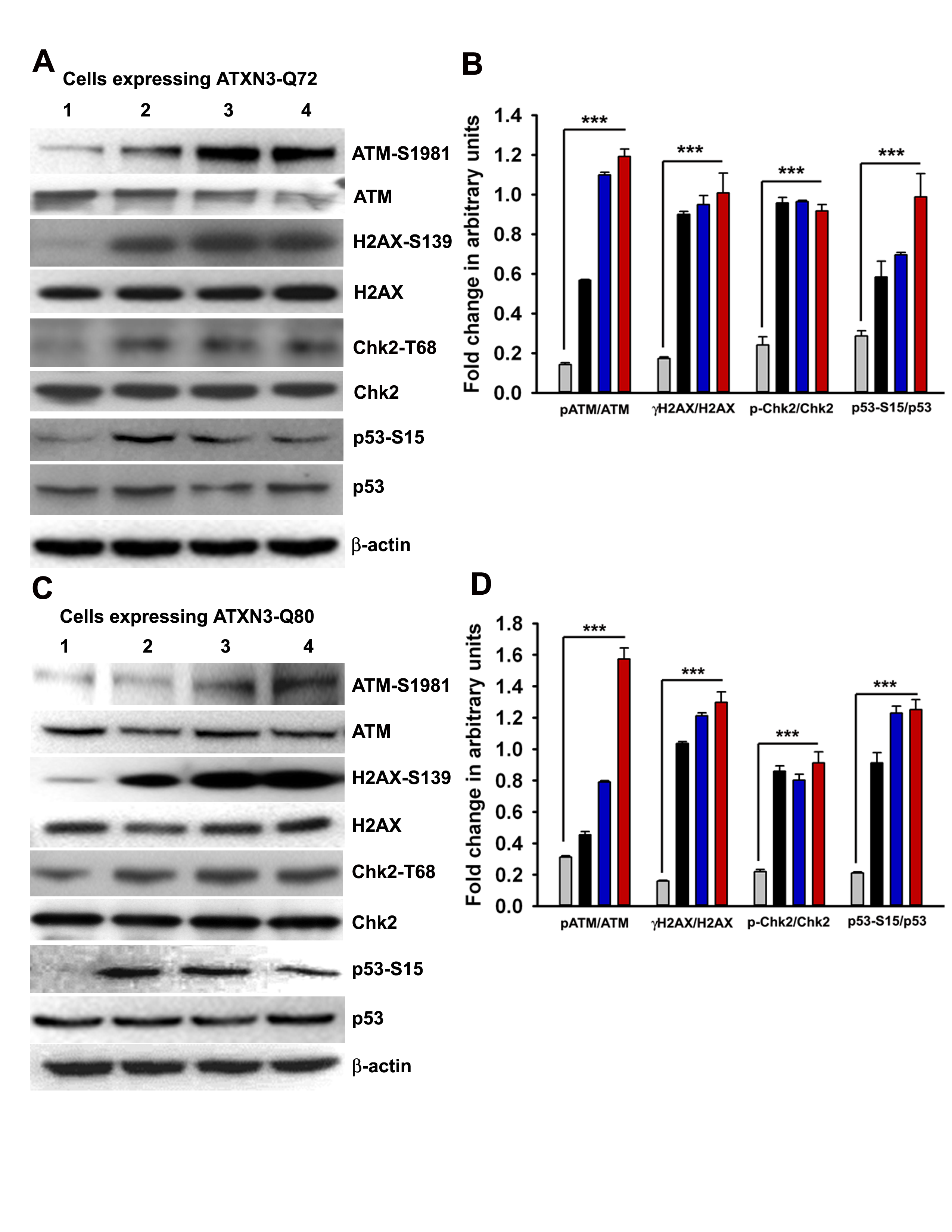

Supplement: S8 Fig — (A) Expression of ATXN3-Q72 was induced in SH-SY5Y cells and cells were harvested 0, 3, 6 and 12 days post-induction (lanes 1 to 4); cell lysates were analyzed by Western blotting to determine the levels of ATM-S1981, total ATM, γH2AX-S139, total H2AX, Chk2-T68, total Chk2, p53-S15 and total p53; β-actin was used as a loading control in A and C. (B) Relative levels of ATM-S1981, γH2AX-S139, Chk2-T68 and p53-S15 with respect to corresponding total protein in cells expressing ATXN3-Q72. Cells were harvested after 0 (grey), 3 (black), 6 (blue) and 12 (red) days of ATXN3-Q72 expression (n = 3, *** = p < 0.001 in B and D). (C) Expression of ATXN3-Q80 was induced in SH-SY5Y cells; cells were harvested 0, 3, 6 and 12 days post-induction (lanes 1 to 4) and cell lysates analyzed by Western blotting to determine the levels of ATM-S1981, total ATM, γH2AX-S139, total H2AX, Chk2-T68, total Chk2, p53-S15 and total p53. (D) Relative levels of ATM-S1981, γH2AX, Chk2-T68 and p53-S15 with respect to the corresponding total protein in cells expressing ATXN3-Q80, shown as described in B. (TIF) [file pgen.1004834.s008.tif]

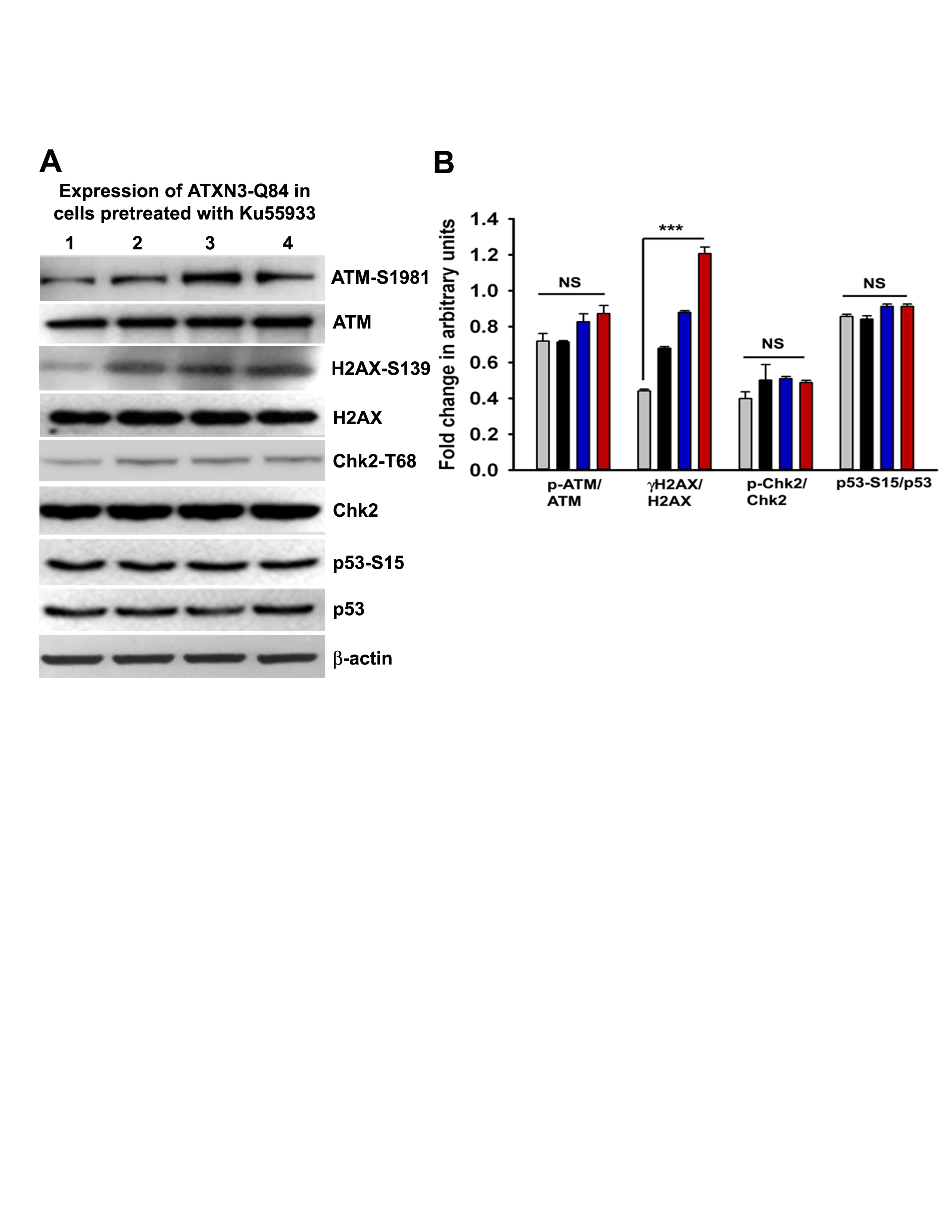

Supplement: S9 Fig — (A) SH-SY5Y cells were differentiated, incubated with Ku55933, and expression of ATXN3-Q84 was induced. Cells were harvested 0, 3, 6 and 12 days post-induction (lanes 1 to 4), and the cell lysates analyzed by Western blotting to detect ATM-S1981, total ATM, γH2AX-S139, total H2AX, Chk2-T68, total Chk2, p53-S15, total p53; β-actin was used as loading control. (B) Relative levels of ATM-S1981, γH2AX, Chk2-T68 and p53-S15 with respect to the corresponding total proteins in cells expressing ATXN3-Q84 and pre-treated with Ku55933. Cells were harvested after 0 (grey), 3 (black), 6 (blue) and 12 (red) days of ATXN3-Q84 expression (data represent mean ± SD, n = 3, *** = p < 0.001). (TIF) [file pgen.1004834.s009.tif]

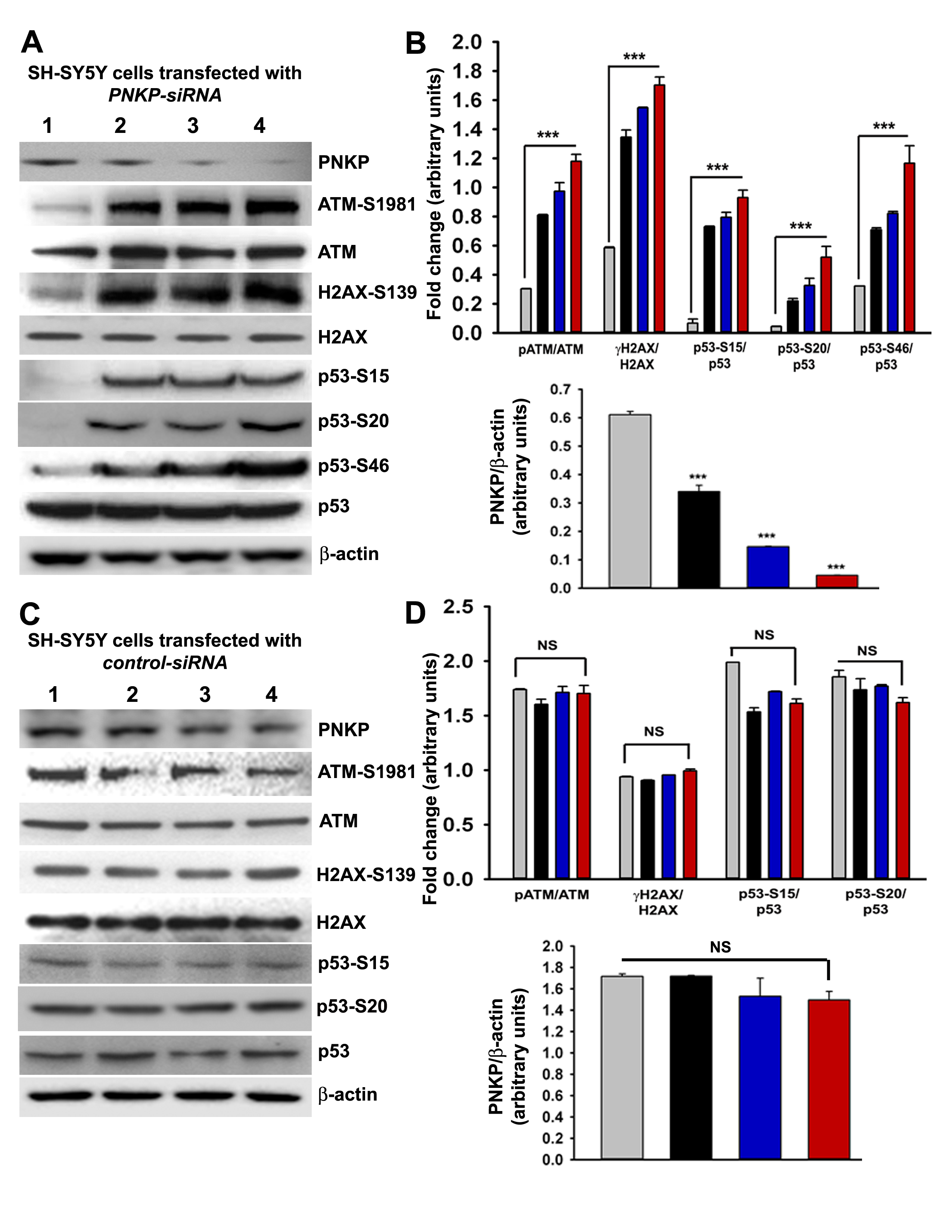

Supplement: S10 Fig — (A) SH-SY5Y cells were differentiated and transfected with 0, 50, 100 and 200 pmoles (lanes 1 to 4) of PNKP-siRNA; 48 hours after transfection, cells were harvested and their lysates analyzed by Western blotting to detect ATM-S1981, total ATM, γH2AX-S139, total H2AX, p53-S15, p53-S20, p53-S46 and total p53. β-actin was used as a loading control in A and C. (B) Relative levels of ATM-S1981, γH2AX-S139, p53-S15, p53-S20, p53-S46 with respect to the corresponding total proteins in cells transfected with 0 (grey), 50 (black), 100 (blue) and 200 (red) pmoles of PNKP-siRNA (upper panel). PNKP level normalized to β-actin is shown in lower panel; n = 3, data represent mean ± SD, *** = p < 0.001 in B and D. (C) SH-SY5Y cells were differentiated and transfected with 0, 50, 100 and 200 pmoles (lanes 1 to 4) of control-siRNA; 48 hours after transfection, cells were harvested and the cell lysates analyzed by Western blotting as in A. (D) Relative levels of ATM-S1981, γH2AX-S139, p53-S15, p53-S20 with respect to the corresponding total protein in cells transfected with 0 (grey), 50 (black), 100 (blue) and 200 (red) pmoles of PNKP-siRNA (upper panel). PNKP levels normalized to β-actin are shown in the lower panel. (TIF) [file pgen.1004834.s010.tif]

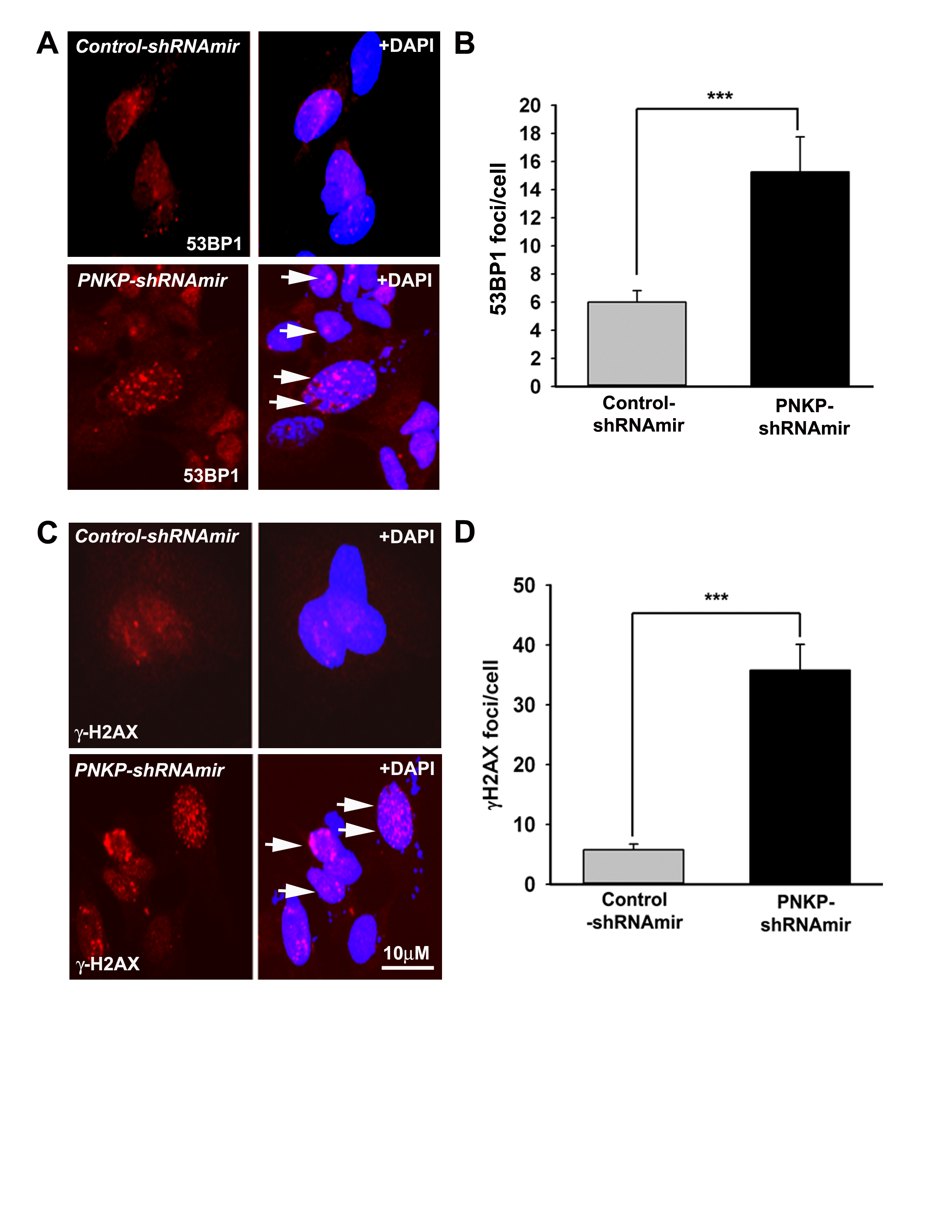

Supplement: S11 Fig — (A) SH-SY5Y cells were transfected with plasmids expressing PNKP-shRNAmir or control-shRNAmir and the transfected cells analyzed by immunostaining with anti-P-53BP1-S1778 antibody (red); 53BP1 foci are shown by arrows. (B) Relative number of 53BP1 foci in SH-SY5Y cells transfected with plasmids encoding control-shRNAmir (upper panel) vs. PNKP-shRNAmir (lower panel). The data represent mean ± SD, n = 100, ***p = 0.001 in B and D. (C) SH-SY5Y cells were transfected with plasmids expressing either PNKP-shRNAmir or control-shRNAmir and the cells analyzed by immunostaining with anti-γH2AX-S139 antibody (red); γH2AX foci are shown by arrows. (D) Relative number of P-γH2AX foci in SH-SY5Y cells transfected with plasmids expressing either control-shRNAmir or PNKP-shRNAmir. (TIF) [file pgen.1004834.s011.tif]

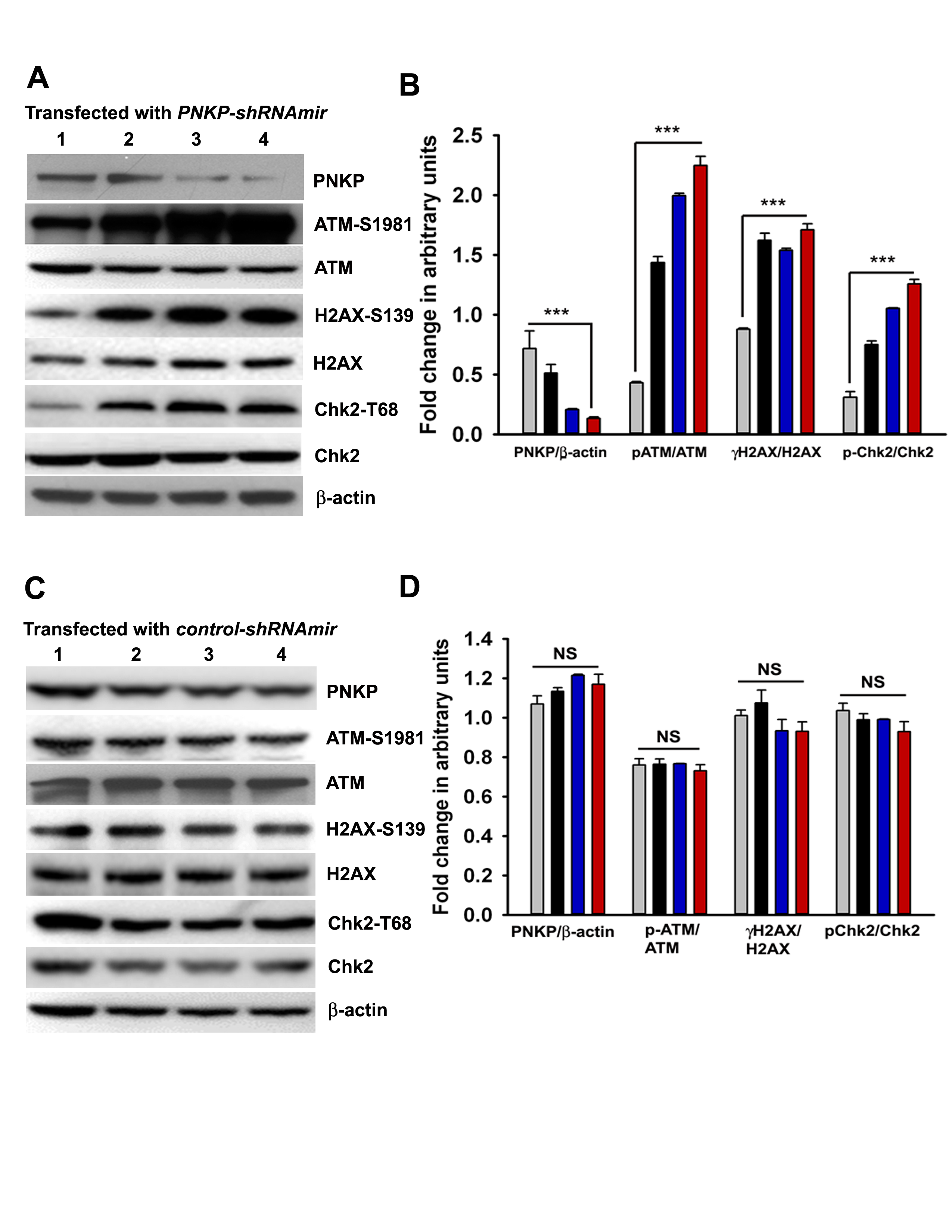

Supplement: S12 Fig — (A) SH-SY5Y cells were transfected with 0, 2, 4 and 8 µg (lanes 1 to 4) of plasmid DNA expressing PNKP-shRNAmir; 48 hours post-transfection the cells were harvested and their lysates analyzed by Western blotting to determine PNKP, ATM-S1981, total ATM, γH2AX-S139, total H2AX, Chk2-T68 and total Chk2 levels; β-actin was used as loading control in A and C. (B) Relative levels of ATM-S1981, γH2AX-S139, Chk2-T68 with respect to the corresponding total protein in SH-SY5Y cells transfected with plasmid expressing PNKP-shRNAmir. Cells were transfected with 0 (grey bar), 2 (black bar), 4 (blue) and 8 µg (red) of plasmid expressing PNKP-shRNAmir (n = 3, data represent mean ± SD; *** = p < 0.001 in B and D). (C) SH-SY5Y cells were transfected with 0, 2, 4 and 8 µg (lanes 1 to 4) of plasmid DNA expressing control-shRNAmir; 48 hours post- transfections the cells were harvested and their lysates analyzed by Western blotting as in A. (D) Relative levels of ATM-S1981, γH2AX-S139, Chk2-T68 with respect to the corresponding total protein in SH-SY5Y cells transfected with plasmid expressing control-shRNAmir. (TIF) [file pgen.1004834.s012.tif]

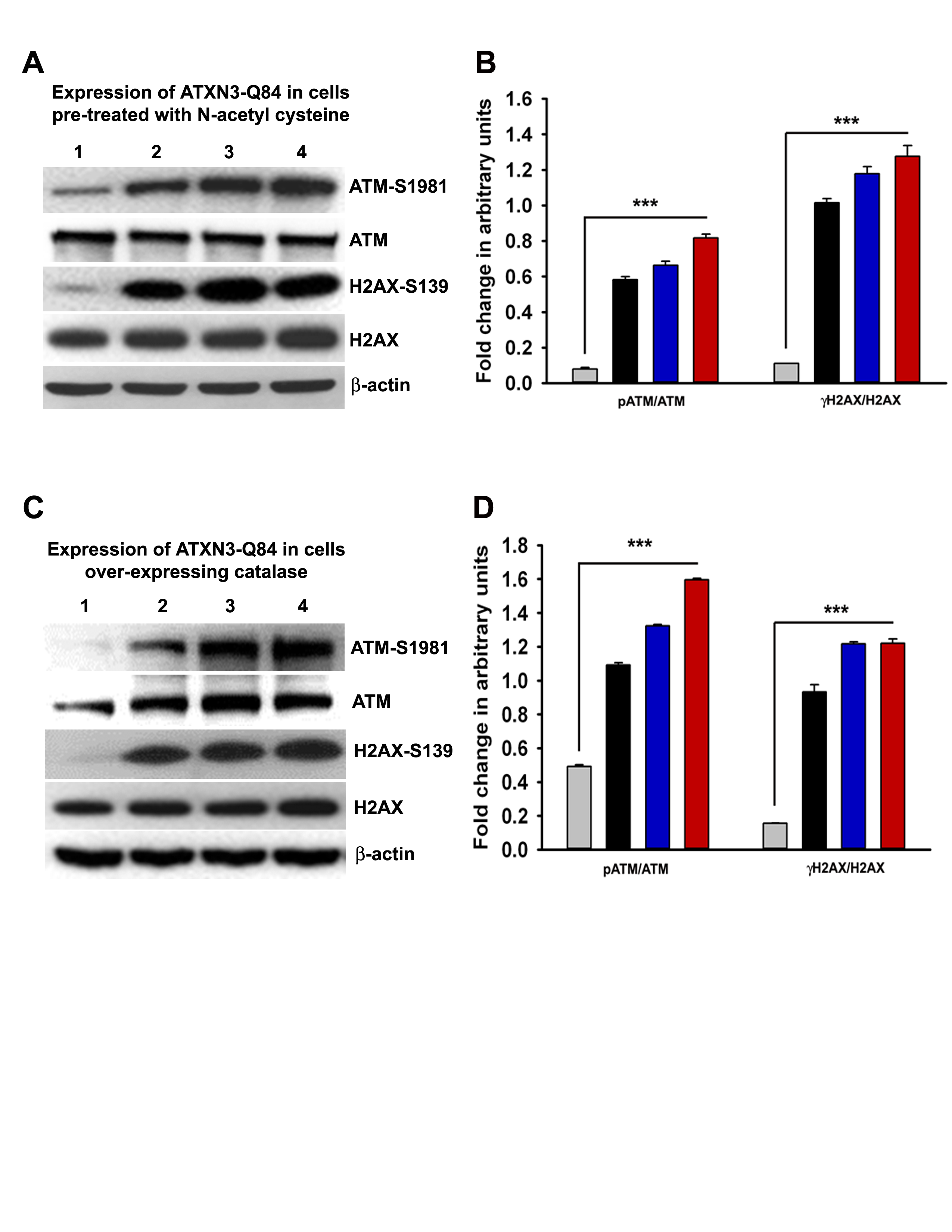

Supplement: S13 Fig — (A) SH-SY5Y cells were differentiated, incubated with N-acetyl cysteine, and the expression of ATXN3-Q84 induced. Cells were harvested 0, 3, 6 and 12 days after induction (lanes 1 to 4), and their lysates analyzed by Western blotting to detect ATM-S1981, total ATM, γH2AX-S139 and total H2AX levels; β-actin was used as a loading control in A and C. (B) Relative levels of ATM-S1981 and γH2AX-S139 with respect to the corresponding total protein determined in the Western blots described in A. Cells were harvested after 0 (grey), 3 (black), 6 (blue) and 12 (red) days of ATXN3-Q84 expression in B and D; n = 3; data represent mean ± SD, *** = p < 0.001 in B and D. (C) Expression of ATXN3-Q84 was induced in SH-SY5Y cells overexpressing the antioxidant enzyme catalase; cell lysates were analyzed as in A. (D) Relative levels of ATM-S1981 and P-γH2AX-S139 with respect to the corresponding total protein were determined in Western blots described in C. (TIF) [file pgen.1004834.s013.tif]

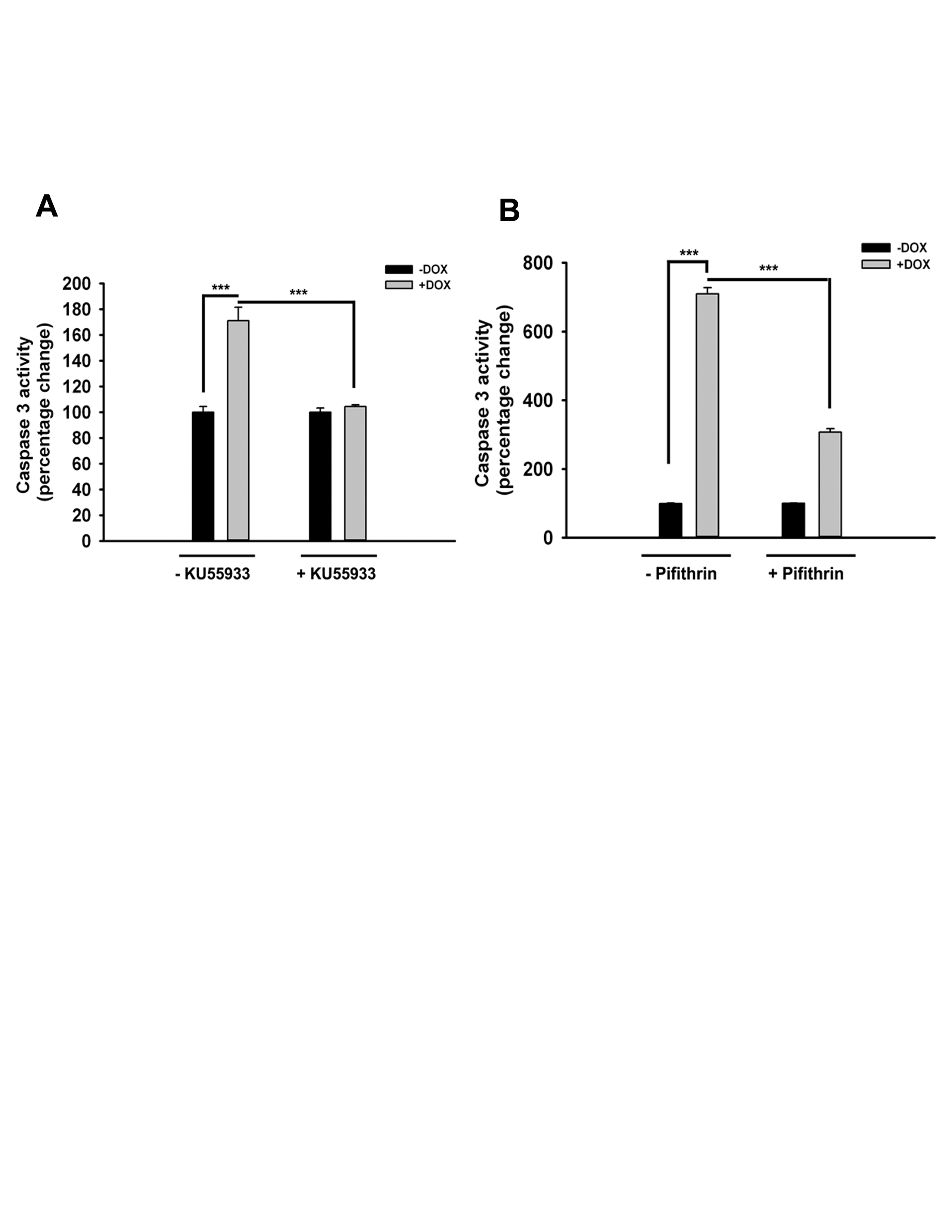

Supplement: S14 Fig — (A) Caspase-3 activities in SH-SY5Y cells expressing ATXN3-Q84 measured before and after treatment with ATM inhibitor Ku55933. (B) Caspase-3 activities in SH-SY5Y cells expressing ATXN3-Q28 and ATXN3-Q84 were measured before and after treatment with the p53 inhibitor Pifithrin-α. Caspase-3 activities are expressed as percentage change normalized to control, and data represent means ± SD; (n = 3; *** = p < 0.001 in A and B). (TIF) [file pgen.1004834.s014.tif]

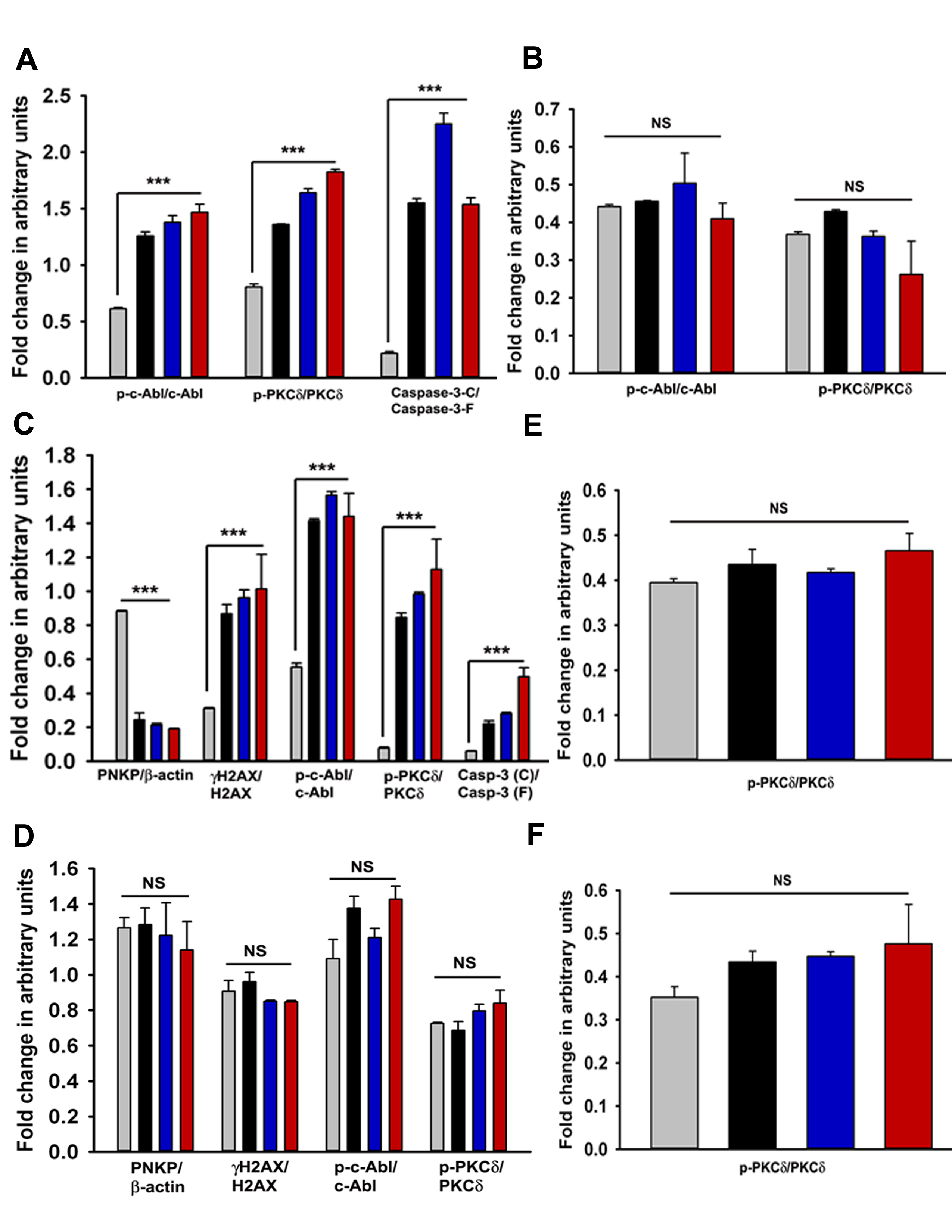

Supplement: S15 Fig — (A) Relative levels of c-Abl-T735, PKCδ-T311 and cleaved caspase-3 (caspase-3-C) with respect to the corresponding total protein in SH-SY5Y cells expressing ATXN3-Q84 (n = 3; data represents mean ± SD, *** = p < 0.001 in A to F). Cells were harvested after 0 (grey), 3 (black), 6 (blue) and 12 (red) days of ATXN3-Q84 expression in A and B. (B) Relative c-Abl-T735 and PKCδ-T311 levels in SH-SY5Y cells expressing ATXN3-Q84 and pre-treated with Ku55933. (C) Relative γH2AX-S139, c-Abl-T735, PKCδ-T311, and cleaved caspase-3 levels with respect to total protein in SH-SY5Y cells transfected with 0 (grey), 50 (black), 100 (blue) and 200 (red) pmoles of PNKP-siRNA. (D) Relative γH2AX-S139, c-Abl-T735, PKCδ-T311, and cleaved caspase-3 levels in SH-SY5Y cells transfected with 0 (grey), 50 (black), 100 (blue) and 200 (red) pmoles of control-siRNA. (E) Relative PKCδ-T311 with respect to total PKCδ in cells pre-treated with STI-571 and expressing ATXN3-Q84. Cells were harvested after 0 (grey), 3 (black), 6 (blue) and 12 (red) of expression. (F) Relative PKCδ-T311 with respect to total PKCδ in cells pre-treated with STI-571 and transfected with 0 (grey), 50 (black), 100 (blue) and 200 (red) pmoles of PNKP-siRNA. (TIF) [file pgen.1004834.s015.tif]

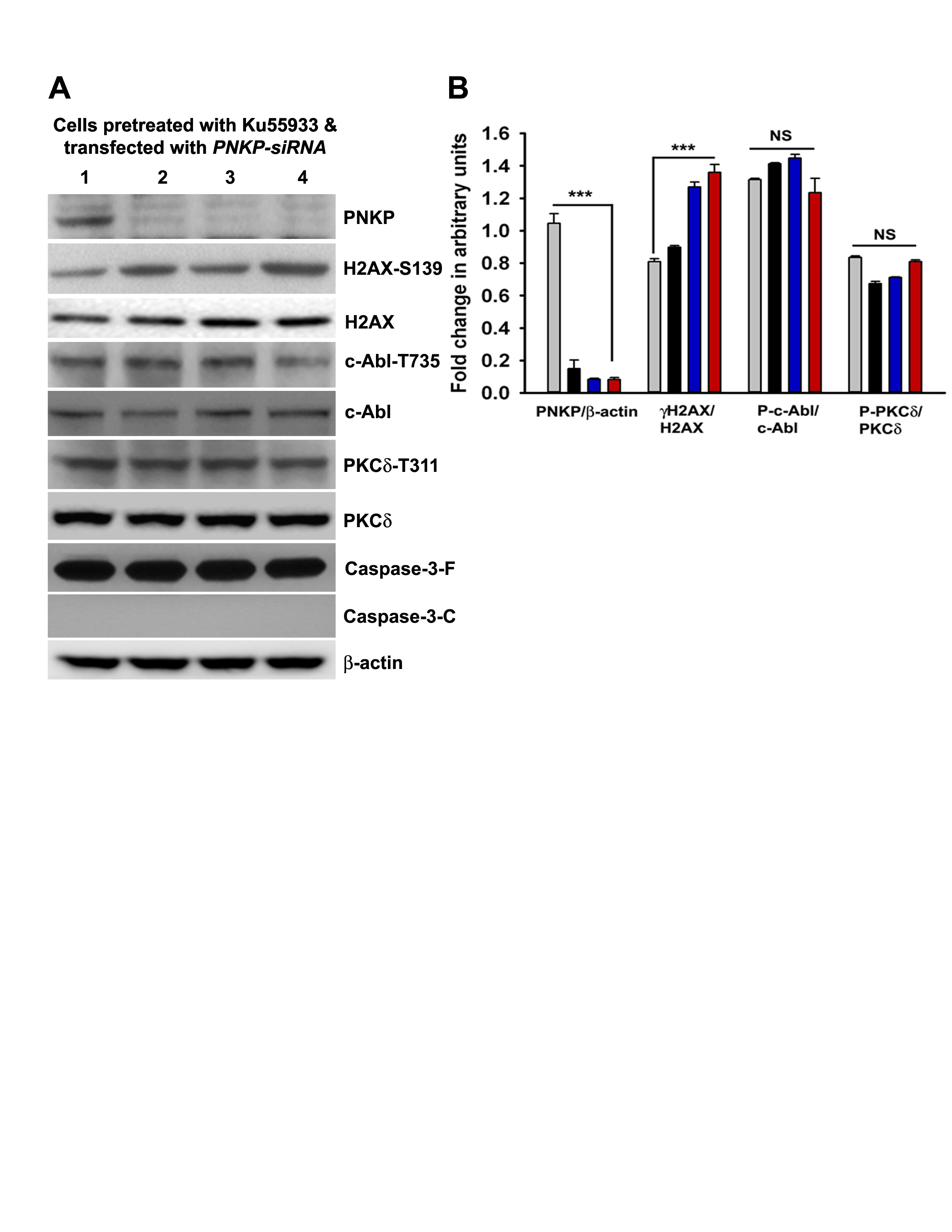

Supplement: S16 Fig — (A) SH-SY5Y cells were differentiated, incubated with Ku55933, transfected with 0, 50, 100 and 200 pmoles of PNKP-siRNA (lanes 1 to 4), and cell lysates analyzed by Western blotting to determine PNKP, γH2AX-S139, total H2AX, c-Abl-T735, total c-Abl, PKCδ-T311, total PKCδ, Caspase-3 (cleaved), caspase-3 (full-length); β-actin was used as loading control. (B) Relative levels of γH2AX-S139, c-Abl-T735, PKCδ-T311, cleaved caspase-3 with respect to the corresponding total protein in cells pre-treated with Ku55399 and transfected with 0 (grey), 50 (black), 100 (blue) and 200 (red) pmoles of PNKP-siRNA. PNKP levels were normalized to β-actin. Data represents mean ± SD, n = 3, *** = p < 0.001. (TIF) [file pgen.1004834.s016.tif]

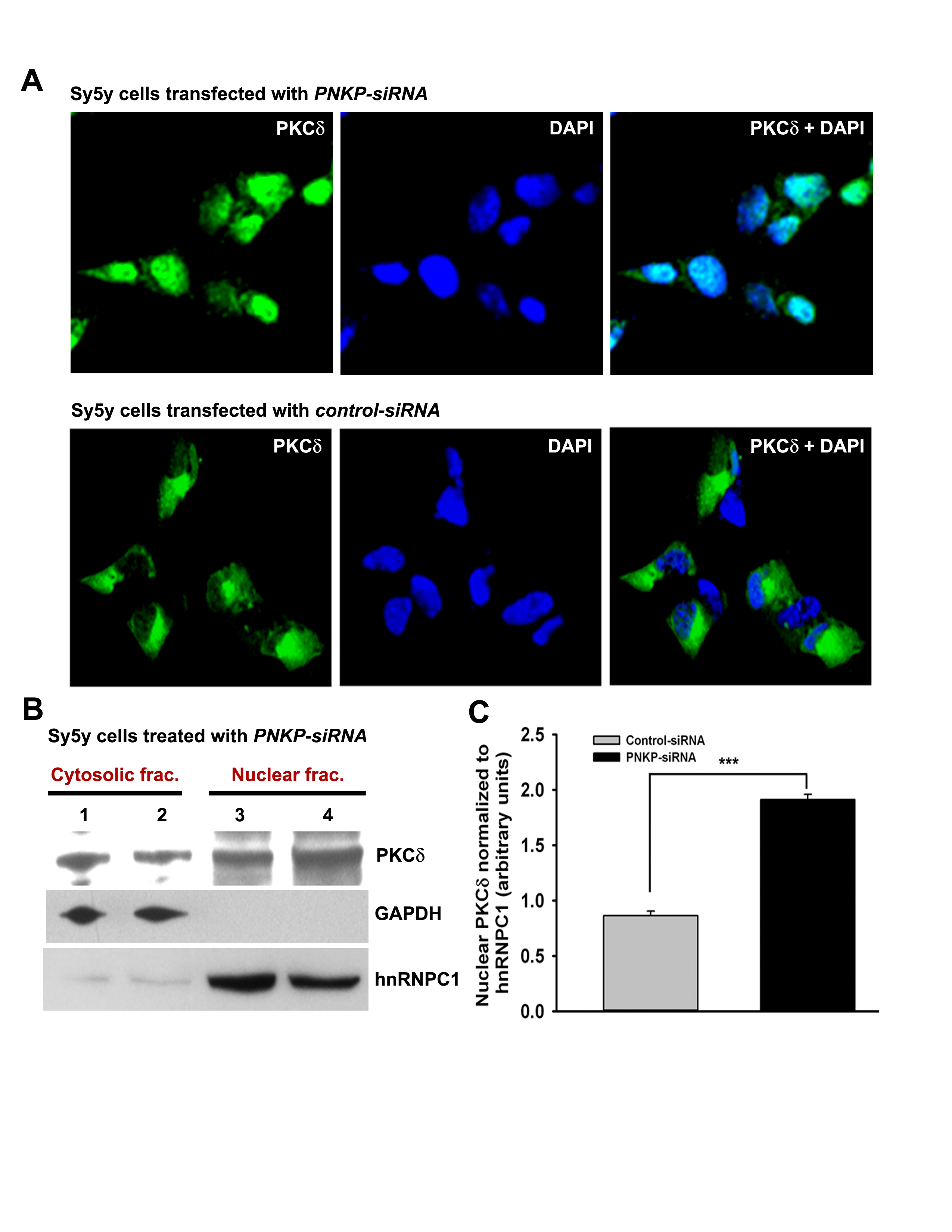

Supplement: S17 Fig — (A) SH-SY5Y cells were transfected with PNKP- or control-siRNA and the transfected cells were immunostained with anti-PKCδ antibody (green). The subcellular distribution of PKCδ was assessed by confocal microscopy; nuclei were stained with DAPI. (B) Nuclear and cytosolic protein fractions were isolated from SH-SY5Y cells transfected with PNKP- or control-siRNA and analyzed by Western blotting with anti-PKCδ antibody to determine the relative nuclear/cytosolic abundance of PKCδ; GAPDH and hnRNPC1 were used as cytosolic and nuclear markers, respectively. (C) Relative levels of PKCδ in cytosolic and nuclear protein fractions in cells transfected with PNKP-siRNA vs. control-siRNA. Data represents mean ± SD (n = 4; *** = p < 0.001) (TIF) [file pgen.1004834.s017.tif]

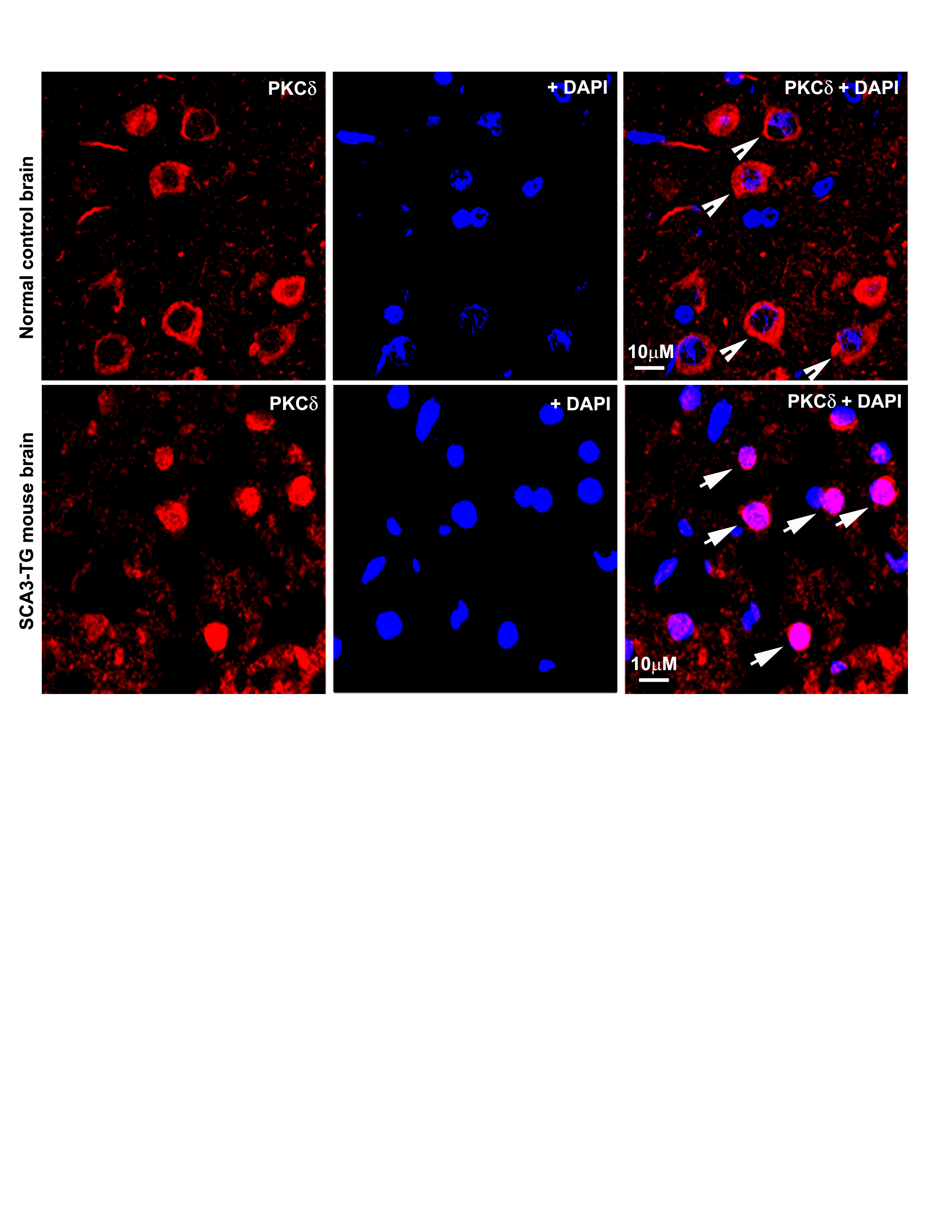

Supplement: S18 Fig — Brain sections from the SCA3 transgenic mouse expressing mutant ATXN3-Q135 (CMVMJD135 mice) and control mouse were analyzed by immunostaining with anti-PKCδ antibody (red) to assess the sub-cellular distribution of PKCδ. Cytosolic presence of PKCδ in the control mouse brain sections are shown by arrowhead, and nuclear PKCδ in the transgenic mouse brain sections is shown by arrows; nuclei were stained with DAPI (TIF) [file pgen.1004834.s018.tif]

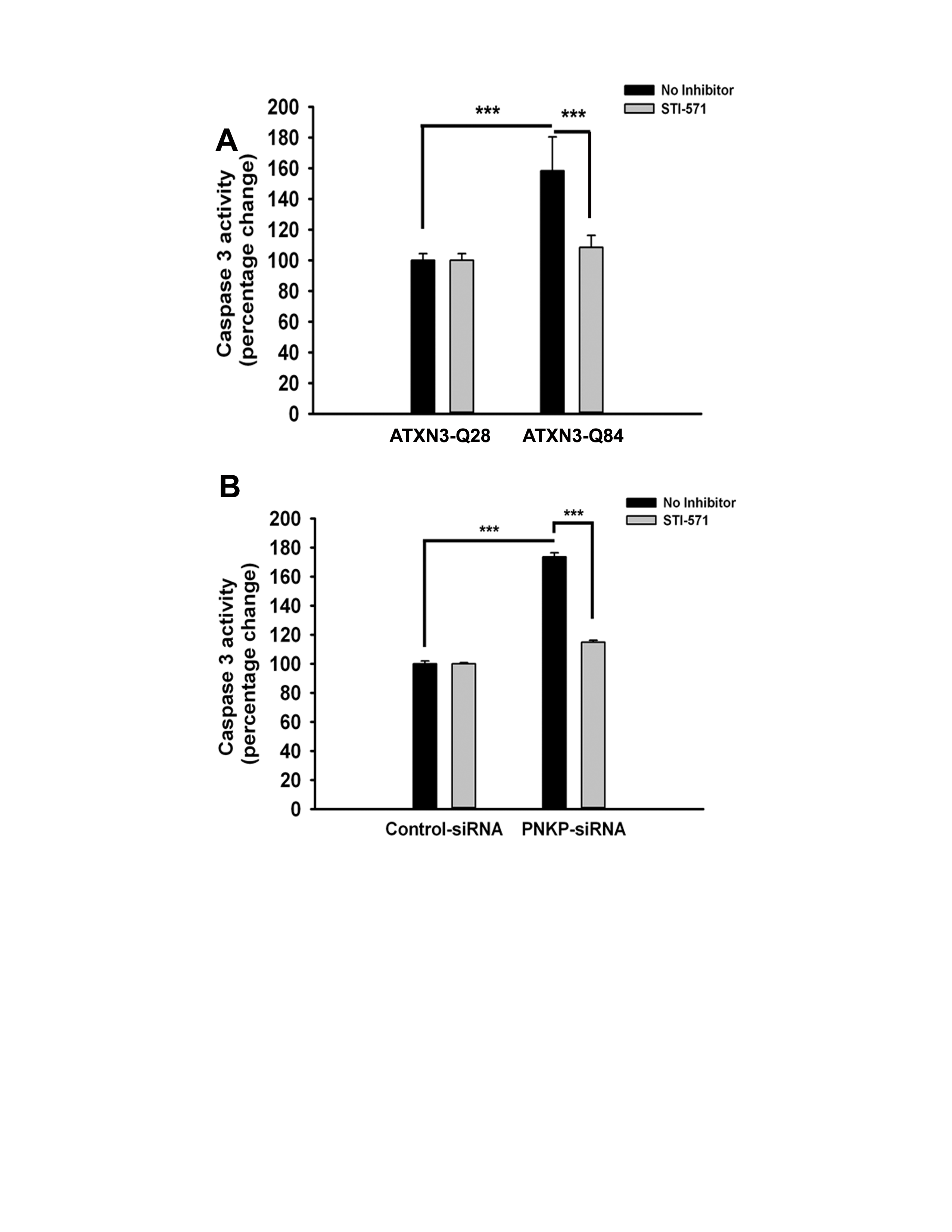

Supplement: S19 Fig — (A) Caspase-3 activities in SH-SY5Y cells expressing ATXN3-Q28 and ATXN3-Q84, and expressing ATXN3-Q28 and ATXN3-Q84 in the presence of STI-571; caspase-3 activities are expressed as percentage change normalized to control. Data represent means ± SD; (n = 3; *** = p < 0.001). (B) Caspase-3 activities in SH-SY5Y cells transfected with PNKP- or control-siRNA, and in SH-SY5Y cells pre-treated with the c-Abl inhibitor STI-571 and transfected with PNKP-siRNA; caspase-3 activities are expressed as percentage change normalized to control. Data represent means ± SD (n = 3; *** = p < 0.001). (TIF) [file pgen.1004834.s019.tif]
